# “Synthetic Map”: A Graphic Organizer Inspired by Artificial Neural Network Paradigms for Learning Organic Synthesis

**DOI:** 10.1021/acs.jchemed.4c00592

**Published:** 2024-09-09

**Authors:** Carlos Luque-Corredera, Elena Bartolomé, Ben Bradshaw

**Affiliations:** † Escola Universitaria Salesiana de Sarrià (EUSS), Pg. Sant Joan Bosco, 74, 08017 Barcelona, Spain; ‡ Institut de Ciència de Materials de Barcelona (ICMAB-CSIC), Campus UAB, Bellaterra 08193, Spain; § Unitat Química Orgànica, Department de Química Farmacèutica de Facultat de Farmàcia, Universitat de Barcelona, 08007 Barcelona, Spain

**Keywords:** Second-Year Undergraduate, Organic Chemistry, Problem Solving/Decision Making, Learning Theories, Organic Synthesis, Neural Networks

## Abstract

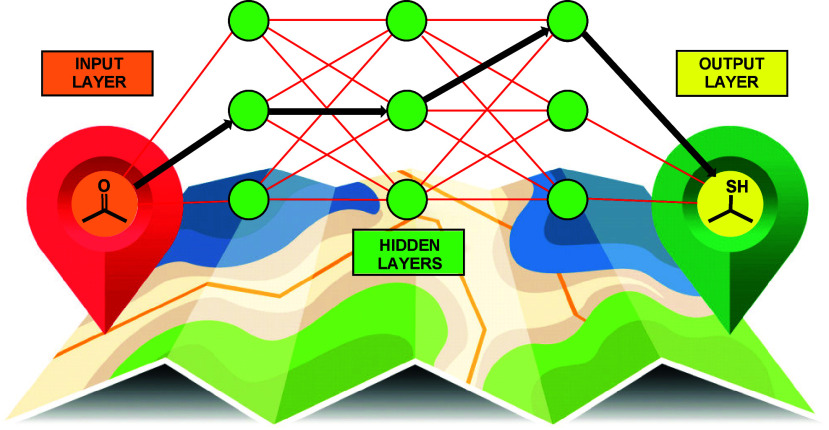

Organic Chemistry is widely recognized as a challenging subject, with the design of syntheses and retrosyntheses identified as particularly difficult tasks. Inspired by the success of artificial neural networks in machine learning, we propose a framework that leverages similar principles to enhance the teaching and learning of organic synthesis. In this paper, we introduce a novel teaching tool, the “Synthetic Map”, that attempts to visually recreate an expert’s mental map and conceptual understanding of organic synthesis built over years of experience. The educational benefits of the Synthetic Map were evaluated through its implementation in an Organic Chemistry course of a Pharmacy degree over two years. The new tool promoted students’ learning by providing a mental organizer fostering a deeper understanding of the subject and empowering students to design and execute effective synthetic strategies.

## Introduction

Organic Chemistry has a long-lasting reputation as a challenging subject.^1−8^ One of the most difficult aspects is designing a synthesis and retrosynthesis.^9−11^ To solve a synthesis problem, students must design a series of reactions combining molecules to give a desired, more complex molecule.^9^ In doing so, undergraduate students often struggle with various challenges. Students must remember the numerous functional groups and the hundreds of essential reactions constituting the organic chemistry canon from memory. They must recall, use, and integrate knowledge and skills learned in previous courses. Often, a significant gap in the teaching of organic synthesis is observed, as students are typically taught reactions, and then they are expected to propose a complete synthesis. Some of the abilities required to plan a synthesis successfully (the “remember” and “understand” skills) can be classed at the lower end of Bloom’s revised taxonomy,^12^ while others (the “synthesize” and “evaluate” tasks) require higher order thinking skills.^9^ Furthermore, a study using Perry’s cognitive science framework highlighted the significant gap between the level of epistemological development most students possess when learning organic chemistry and the level of sophistication required by the subject. Particularly problematic are “dualistic thinkers” who search for a *unique correct answer*, while organic chemistry frequently requires students to find *several* correct answers or devise *multiple* reaction pathways.^13^

Various educational materials and learning activities have been proposed to address these challenges in recent years. These include card games for learning functional group transformations^14^ or synthetic problems,^15^ gaming Apps^16^ for digital platforms, and synthetic challenges facilitated by artificial intelligence (AI)-based organic synthesis software.^17^ Some textbooks have introduced strategies for solving synthesis problems,^18−20^ and incorporated illustrative case studies to motivate learning by contextualizing organic reactions.^21,22^ To the same end, “road map” problems have been developed, requiring students to “fill in the blanks” in the synthesis of target compounds.^23^ Learning activities that provide scaffolding and feedback have also proven beneficial for organic synthesis learning and developing critical-thinking skills.^24−26^ Furthermore, some authors have investigated how students approach these synthesis learning activities and whether they do as intended by the teacher to overcome the barriers to learning.^9,27^

However, presenting theoretical information and reactions to students has seen limited progress. Some authors have suggested teaching mechanisms and electron-pushing formalism before reactions.^28^ Nevertheless, most organic chemistry textbooks^29^ and curricula still revolve around individual functional groups and their related chemical reactions, making it difficult for students to recognize connections between different functional groups and reactions. This leads to their mental map consisting of disconnected and fragmented elements of information hindering combination into a synthesis. In the realm of organic chemistry, a complex field that demands proficiency in a diverse range of concepts and skills with interconnected ideas, cognitive concept maps have emerged as valuable tools to scrutinize how students organize and interrelate concepts across various topics.^30−34^ Findings indicate that organic students often struggle to view the field as a coherent whole, instead taking the perspective that each reaction, its reagents, and its mechanism must be memorized individually.^1^ In particular, several published studies have reported the students’ fragmented understandings of the concepts of nucleophiles/electrophiles^35,36^ and leaving groups,^37^ which are pivotal for mastering reactions.^28^ According to Ausubel and Novak’s Human Constructivism theory, students’ fragmented knowledge structures, often stemming from rote memorization, can impede their capacity to engage in “meaningful learning”.^38,39^ D.P. Ausubel underscored the importance of the student’s cognitive structure in determining the potential meaningfulness of new information,^40^ and advocated for the use of “organizers” prior to exploring unfamiliar material to facilitate meaningful learning and retention.^41^

In contrast, teachers have a richer mental map and more interconnected understanding of organic chemistry, gained through specialization, many years of preparation of teaching material, research activities, all combined with a natural aptitude for the subject. For example, a comparison between expert and novice knowledge structures about nucleophiles and electrophiles revealed the much more interconnected cognitive maps of the former.^36^ This holistic comprehension allows instructors to perceive relationships and patterns and solve synthesis problems more easily.

Herein, we propose using concepts of Artificial Neural Networks (ANNs) to explain the key differences in mental structure between beginners of an organic chemistry course and seasoned experts and develop a visual tool to bridge the gap between the two.

**Figure 1:**
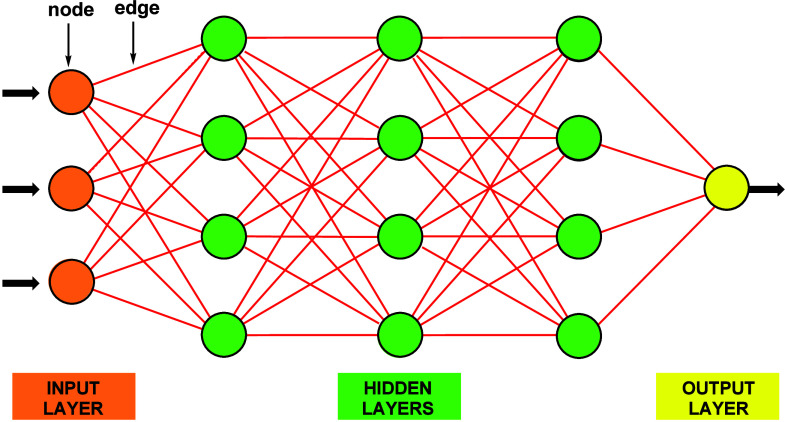
Representation of an artificial neural network (ANN): applied to organic synthesis, the input layer nodes (orange) represent the starting materials; the output layer represents the target molecule (yellow); and the edges (red line interconnections) represent synthetic reactions.

An artificial neural network (ANN) is a computational model inspired by the structure and functioning of biological brains.^42,43^ It consists of interconnected nodes (neurons) arranged in layers; each unit receives inputs, applies a mathematical operation, and generates an output signal, which serves as input for the subsequent layer. The layers between the input and output layers through which the network travels are called hidden layers (Figure 1). Utilizing machine learning principles, ANNs can learn from data and make predictions.^44^ Applied to organic synthesis, the network’s input layer represents our possible starting materials. The output layer represents our target molecule (functional group). The edges are chemical reactions (reagents required to enact the chemical transformation), and the hidden layers represent the intermediate functional groups we must pass through to reach the desired solution.

**Figure 2:**
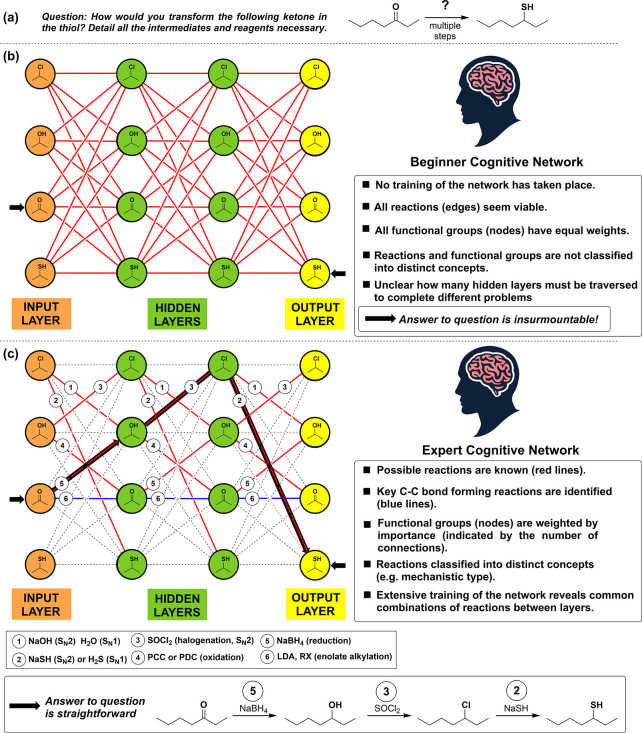
ANN approach for solving an (a) organic synthesis problem, (b) underlying cognitive thinking process for a beginner, and (c) underlying cognitive thinking process for an expert, along with a solution to the problem.

To illustrate the critical differences in the mental organization of the beginner and the expert in practicing organic synthesis, we can use ANN diagrams to show how they would each approach solving an exercise such as that presented in Figure 2a, in which a ketone (3-heptanone) must be converted to its thiol derivative. While an ANN to solve this problem should incorporate all functional groups and multiple hidden layers, for illustration, we will limit our ANN to just four functional groups and two hidden layers necessary to solve the problem in question (see Figure 2b). With the network constructed, we can see the daunting challenge novice students face when confronting a synthesis problem in their mental representation (Figure 2b). For the beginner for whom no training of the network has taken place, (i) all reactions seem viable, (ii) all functional groups (nodes) have equal weights, (iii) reactions and functional groups are not classified into distinct concepts, and (iv) it is unclear how many layers must be traversed to arrive at the correct solution.

Now, if we update the same network to reflect that of an expert after many years of training (analogous to the machine learning of ANN), we have the network shown in Figure 2c. We see that the reactions have been learned and classified by reaction type, while routes that are not viable have been closed down and eliminated. We can observe that some nodes have more weight than others, with the alcohol, ketone, and chloroalkane functional groups having more active connections, indicating their higher influence on the network’s decision-making process. In contrast, the thiol group can be observed to have a secondary or negligible contribution to the network.

While this network diagram may help us understand the underlying thinking process of the expert, in practice, the question in Figure 2a is likely tackled more indirectly. First, the question is analyzed and broken into consistent parts (step 4 of Bloom’s taxonomy^12^), resulting in the following observations: (i) No Carbon-Carbon bonds are formed; therefore, only functional group manipulations are required. (ii) The product has a different oxidation state to the starting material; therefore, one step must be a reduction. (iii) Ketones are readily reduced to alcohols. (iii) Thiols are end products, not usually intermediates. (iv) Thiols are formed from haloalkanes via substitution reactions. (v) OH is a poor leaving group, and (vi) Cl is a good leaving group. With all this information in working memory, the expert will then proceed to the “Synthesis” or “Create” phase (stage 5 of Bloom’s taxonomy). Here, all these critical pieces of information are used to generate a solution based on the expert’s unique mental map that will be used intuitively to guide the assembly of the components outlined above into the correct sequential order.

Teaching these more abstract thinking skills is a challenging proposition, since students need to have fully internalized all the information taught in the course. However, with access to the network presented in Figure 2c, solving the problem shown in Figure 2a becomes relatively straightforward for even a beginner with just a basic level of instruction.

However, the ANN presented in Figure 2c represents only a tiny fraction of the total combinations possible in organic synthesis, and adapting the network to solve all synthetic problems is challenging. To make the ANN useful, it should include all functional groups (around 40) encountered in a typical standard undergraduate course. Moreover, for educational purposes, the design of the ANN-based Synthetic Map should be easy to visualize and incorporate ideas from conceptual maps.

Constructing a comprehensive network that includes all relevant functional groups may quickly become confusing, therefore, it is essential to design it adequately to ensure manageability for teaching and learning purposes. While some reaction maps have been developed, found in textbooks^45,46^ and online sources,^47−57^ they mainly focus on individual functional groups, requiring students to piece together separate maps for complex synthesis. Some maps attempt to connect specific functional groups^51,52^ and use color-coded representations for different types of reactions.^53−55^ However, they lack logical visual organization, making them challenging to use and remember. Additionally, they often display limited interrelationships among functional groups, limiting synthetic route possibilities and possibly giving students the wrong impression that there is only one method to synthesize a given molecule. Despite the availability of some of these maps for purchase online, limited research has been conducted to assess their effectiveness as educational tools.^58,59^

In this context, the specific *research questions* of this work are:

- **RQ1**- Can we design a “Synthetic Map” visual tool based on ANN paradigms, recreating the neural network of experts in organic synthesis built over years of experience?
- **RQ2**- Can we use this novel graphic organizer to teach Organic Chemistry and specifically overcome the learning problems often encountered by students planning and performing multistep syntheses?

In the remainder of this paper, we will first outline the design of “Synthetic Map,” an ANN-inspired comprehensive visual tool connecting all functional groups and integrating the necessary information to perform complex organic synthesis. Second, we will describe how this Synthetic Map has been utilized to teach an Organic Chemistry course for two years in the context of a Pharmacy University degree. Finally, we will analyze the impact of incorporating the map on students’ organic synthesis skills and their perception of the subject.

## Methodology

### Participants and Setting

The study was conducted in an Organic Chemistry II course, part of the second-year curriculum in the five-year Pharmacy degree program at the University of Barcelona. Organic Chemistry II is a 6 ECTS-credit course, divided into 48 h of theory, 12 h of tutorials, 30 h of supervised, and 60 of autonomous work, amounting to 150 h. The learning objectives of the course are the following:

- Rationalize organic compounds’ basic reactivity according to their functional groups’ structure.
- Gain global knowledge of the processes involved in the transformations of organic compounds.
- Know the most general aspects of essential organic synthesis.
- Write reasonable mechanisms for the main transformations that affect primary functional groups.
- Use stereochemical considerations when analyzing mechanisms and transformations.

The Organic Chemistry II course is organized in three main thematic blocks:

1. Reactions of compounds with single bonds: 1.1. Organic reactions: general concepts; 1.2. Aliphatic nucleophilic substitution reactions; 1.3. Elimination reactions.
2. Reactions of unsaturated hydrocarbons and aromatic systems: 2.1 Reactions of unsaturated hydrocarbons; 2.2. Reactions of benzene and its derivatives.
3. Reactions of carbonylic compounds: 3.1. Aldehydes and ketones. Nucleophilic addition reactions; 3.2. Carboxylic acids and their derivatives. Substitution reactions in the acyl group; 3.3. Carbonyl compounds. Reactions on the α position and condensation reactions.

**Figure 3:**
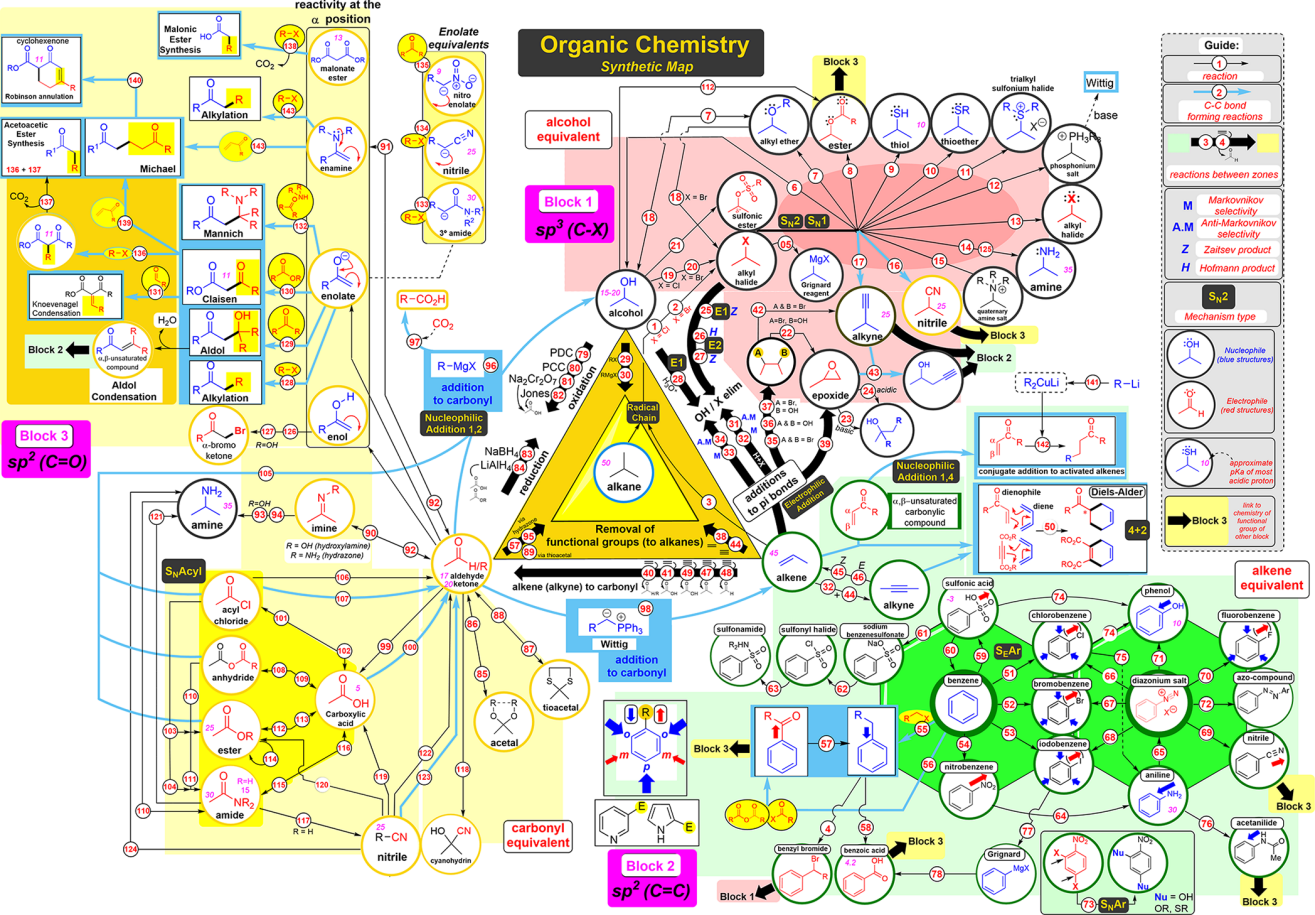
Designed “Synthetic Map” showing the relationship and connectivity between different functional groups. Copyright Dr. Ben Bradshaw (Universitat de Barcelona) 2023.

The course assessment is based on two exams, a partial and a final, where students are asked to solve several synthesis exercises of increasing complexity, involving only one or two reactions, of a complex synthesis route (*vide infra*Figure 3 for some examples).

The “Synthetic Map” was implemented during two academic years, 2021/22 and 2022/23. A single teacher imparted the course, the conceptual designer of the map, with broad teaching and research experience in Organic Chemistry. The cohort of students consisted of 40 students per class. All students had previously taken an introductory course on Organic Chemistry I. About 30% of each group in this study consisted of students who had taken the class before (with another teacher) and were repeating the course.

### Data Collection and Analysis

A voluntary survey was conducted on courses 2021/22 and 2022/23 students. Additional questions were administered to retakers to differentiate the impact of the Synthetic map from the effect of encountering the material for a second time. Furthermore, specific questions were directed to retakers of the 2021/22 academic year who had previously experienced the Organic Chemistry II course before introducing the Synthetic Map to gather their opinion on its benefits. Twenty-five students responded to the survey, including 17 from the 2021/22 course (5 retakers) and 8 from the 2022/23 course (3 retakers). The questionnaire items can be found in the Supporting Information (S3). We also considered the course material (slides and synthetic exercises), exams, and teacher observations throughout the course.

## Results

### Synthetic Map Design

In this section, we describe the design of our ANN-inspired “Synthetic Map”, which was aimed to be used as a central teaching tool in an undergraduate Organic Chemistry course. We delineate the core principles that led to this map structure. However, it is essential to underscore that these same principles can be used to design other ANN-type synthesis maps to meet specific course requirements or levels.

As commented in the Introduction section, a synthetic map resembling Figure 2 would soon become too unwieldy as more functional groups are incorporated. Therefore, we removed redundant elements to make the map less complex and suitable for educational purposes. The Synthetic Map is designed to show only the viable pathways corresponding to known chemical transformations, i.e., all nonviable connections between nodes have been eliminated. Furthermore, each group’s location on the map reflects its importance (weight, in ANN terms): important groups, like alcohols, ketones, and alkenes, with multiple connections are placed closer to the center, while those groups with limited use (e.g., thiols), which are usually end targets in synthesis, are placed toward the peripheries of the map.

The final Synthetic Map design is depicted in Figure 3. It includes three main components: (i) three core functional groups, (ii) three “functional group equivalents”, and (iii) the C–C bond-forming reactions. The Organic Chemistry course encompasses a vast array of functional groups, and memorizing them all and understanding their reactivity can be challenging. The Synthetic Map simplifies this complexity by focusing on three core functional groups: alcohols, alkenes, and carbonyls (aldehydes or ketones), which were deemed the most important based on one of the author’s (B.B) extensive research experience in synthetic organic chemistry. These key functional groups are positioned at the corners of a central triangle on the map.

Each core functional group is positioned adjacent to a colored zone called “functional group equivalents”. Each of these regions contains functional groups that share similarities in terms of structure and reactivity with the core functional groups and has a correspondence with one of the thematic blocks covered in a typical organic chemistry course:

- Block 1 (salmon pink region) - Alcohol equivalents: sp^3^ hybridized functional groups
- Block 2 (green area) - Alkene equivalents: carbon–carbon sp and sp^2^ hybridized functional groups
- Block 3 (yellow area) - Carbonyl Equivalents: sp^2^ hybridized carbon–heteroatom functional groups

Colors and shapes were used to highlight conceptual groups of reactions. It should be noted that the images used for each functional group in the map are conceptual only; the position of the group in the molecule and the number of carbon atoms will vary from case to case and are not meant to be literal.

Arrows represent the edges between the ANN nodes in the map (reactions). Black arrows are used exclusively to denote the “interconversion of functional groups,” so they do not encompass transformations involving carbon-carbon bond formation, which are instead represented by blue arrows. This distinction highlights the importance of carbon-carbon bond-forming reactions in constructing the carbon skeleton of molecules. The small numbers in the map direct to a list of essential reactions (see S1). Physical A3 copies of the map were distributed at the beginning of the course, and a digital version was made available on the learning platform, www.syntheticmap.com, allowing students to access additional details about reactions (for example, reaction overview and detailed mechanism) by clicking on the numbered links within the small blue circles.

Having presented this cognitive mental framework to students, it was required to “train” them in its use to make this knowledge their own. By giving the student a clear cognitive map, dominant mental pathways are highlighted, removing the need to discover these connections through years of study and providing them with a “scaffold” to incorporate new information and concepts.

### Organic Chemistry Course Organization Based on the Synthetic Map

In this section, we elaborate on how the Synthetic Map was utilized to structure and integrate the entire Organic Chemistry II course. The map served as a unifying tool to connect various course themes and facilitate the instruction of synthetic reactions to students.

#### Presenting the Map

Based on our experience, the most effective way to introduce the tool is by first providing a brief overview of the “Synthetic Map” and its organization. For pedagogical purposes, we explained how to use the Synthetic Map by drawing a parallel to navigating a subway map, something students are already familiar with. Thus, they can think of the Synthetic Map as a tool for making a “synthetic journey,” where you begin at the “initial functional group” (your starting station), aim for the “target group” (your desired destination), and figure out the best route(s) to get there. Continuing with the analogy, sometimes specific routes may be blocked or affected by issues (like unsuccessful reaction sequences). In such instances, the map helps identify alternative paths for synthesis.

Following this introduction, a partial map region is introduced in each class, which is connected to previously studied areas. Sections of the map are then systematically added clockwise, gradually introducing all the groups in each of the three blocks of the course. This approach helps students narrow their focus, understand how new reactions and concepts are interconnected to previously covered reactions, and help securely interconnect the different concepts in their minds.

Given that most reagents are petrochemical derivatives, an excellent place to start is alkanes, located at the center of the map, and how they are converted to haloalkanes. This naturally leads to discussing these compounds’ use in nucleophilic substitution reactions, which allow access to other compounds with *sp*^*3*^ hybridization, such as alcohols, thiols, ethers, nitriles, etc. This, in turn, leads to the introduction of elimination reactions (E1 and E2), connecting us to another significant section of the map: alkenes. The next step is the introduction of electrophilic addition reactions, which allow access to compounds from the first region, including alcohols and haloalkenes. Using the map at each stage makes it possible to show the relationships between different reactions and reinforce the previously covered material. For example, elimination and electrophilic addition are two directions of travel between functional groups. The course then progresses with the discussion of aromatic chemistry, oxidation and reduction reactions, carbonyl chemistry reactions (such as additions to carbonyls and carboxylic acid chemistry), and finally, the reactivity of carbonyls as nucleophiles with Aldol-type reactions.

#### Training the Neural Network Using the “Synthetic Map”

**Figure 4:**
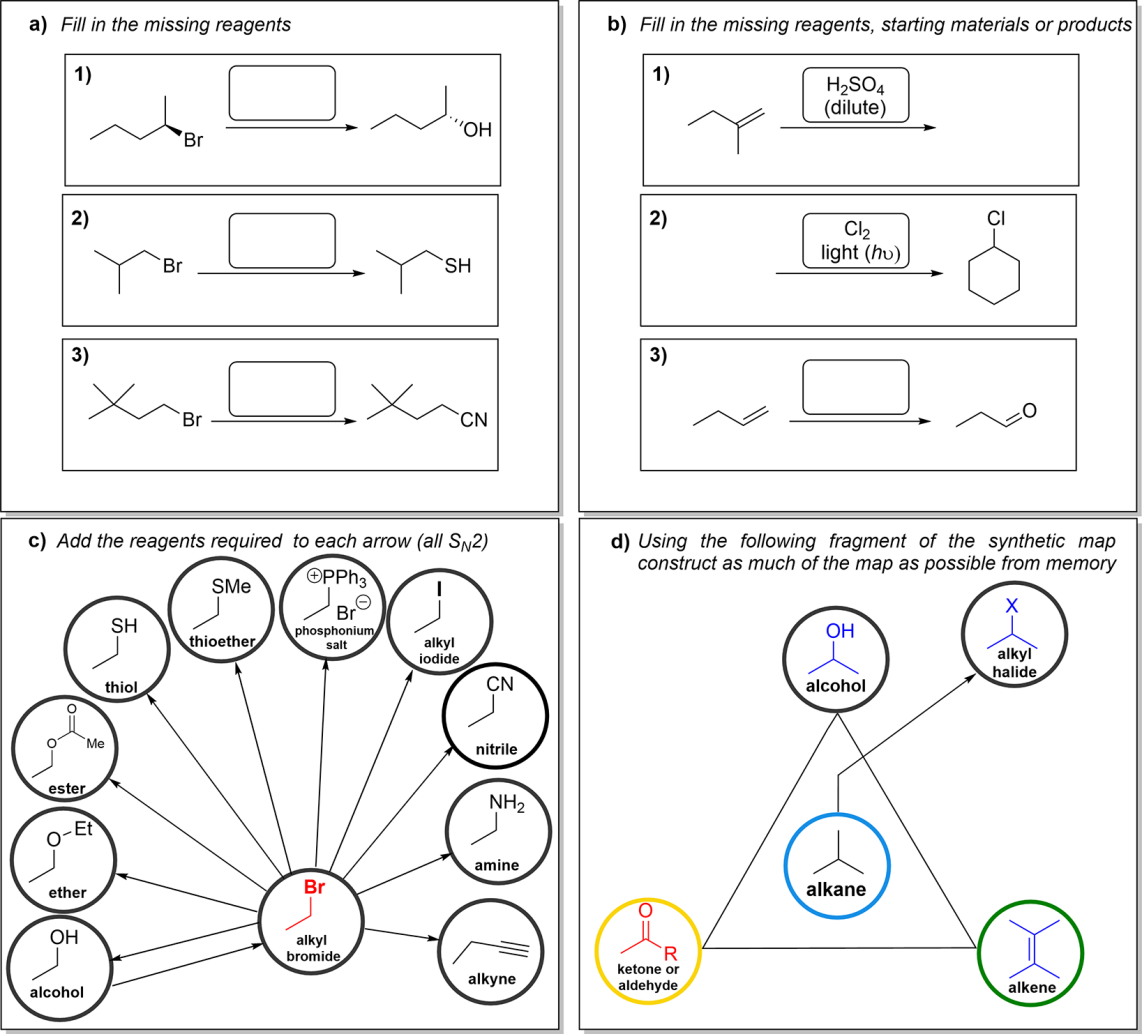
Training of the neural network: characteristic examples of synthetic exercises of gradually increasing difficulty employed to learn using the ANN “Synthetic Map”. (a) A simple, one-step transformation exercise with the student required to fill in the reagents necessary. (b) A slightly more complex one-step transformation exercise, where either the initial starting material, the reagent, or the final product is missing, and the reactions are presented in random order. (c) Exercise where students must fill in the reagents on the part of the map corresponding to the section that has just been studied. (d) Exercise where students are guided through recreating part of the map by hand/freehand drawing.

The map was shown at the beginning of every lecture to introduce the reactions studied during the session. It was also utilized to solve synthesis questions arising throughout the course. This constant visual exposure was used to promote subconscious learning and help students structure the material over the course. Furthermore, to develop students’ organic synthesis skills, we proposed solving different types of problems with an increasing degree of complexity. A list of representative exercises is included in the Supporting Information (S2). These exercises may be categorized into different types, as illustrated by the archetypal examples depicted in Figure 4.

1) In the most straightforward exercises, students receive a list of reactions and are encouraged to locate them on the map and find the reagents necessary to complete the transformations. These exercises aim to learn reactions and help students fix the location of the information on the map, and they were presented in the order they were covered in the course (Figure 4a).
2) A slightly more complex variation of the previous exercise involves presenting students with a reaction and requiring them to fill in the starting material, reagent, or final product (Figure 4b). The reactions do not necessarily appear in the order presented in the class.
3) In a different type of activity, a selected part of the Synthetic Map was provided with blank spaces to fill in the reagents (Figure 4c).
4) An advanced version of the former asks students to draw different parts of the map. For instance, they were instructed to draw the central triangle and then attempt to add parts from this main structure (see Figure 4d). This exercise aims to solidify students’ mental connections as they recreate the map. As a starting activity, the teacher guided the map reconstruction step by step, verbalizing the thinking process while drawing it on the blackboard.

With the above training completed, the teacher tackled more complex synthesis problems. As before, these problems were presented with increasing difficulty levels to prevent overwhelming the students. This was done by introducing syntheses that required only one intermediate step (i.e., only one hidden layer) and then gradually progressing to cases that needed up to four intermediates. These more advanced exercises were tackled toward the end of the course.

#### Synthetic Routes

We provide examples illustrating how the Synthetic Map was utilized to solve synthetic problems. Typically, students were asked to draw a synthetic scheme for preparing a target compound (the output) from an initial organic compound (the input). Before tackling any problem, students were provided with the following list of items outlining the steps to approach the problem-solving:

1) Identify the functional group of the starting material and the functional group present in the final target compound.
2) Is a carbon–carbon bond formed? This point aims to draw the student’s attention to the types of bonds formed. In forming a C–C bond, the student must incorporate a route that includes a reaction with a blue arrow.
3) Find a possible route between the two functional groups (identified in step 1) and incorporate a C–-C bond forming reaction (blue arrow) if you answered yes to step 2. Once identified, write out the sequence of intermediates required and the reagents required (by consulting the numbers from the Synthetic Map if necessary).

**Figure 5:**
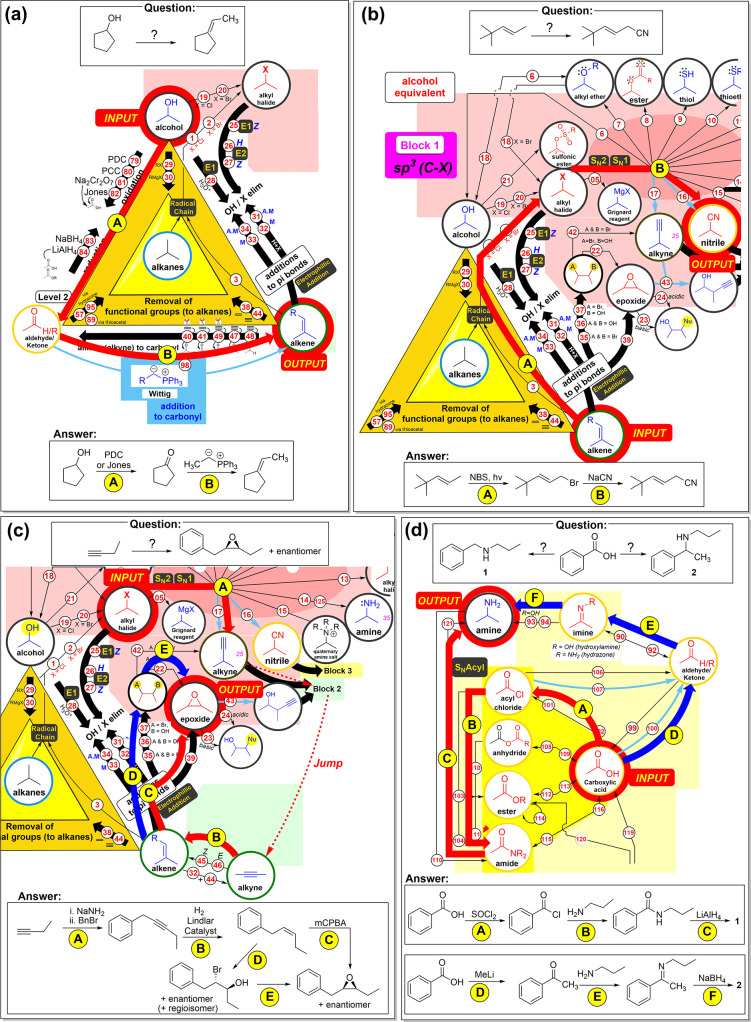
Examples of the “Synthetic Map” to find a “synthetic route” to prepare a target compound from an initial compound. In each example (a–d), we show the problem exercise, the “synthetic route”, and the answer to the question. (a) Example of a linear route around the core triangle to prepare an alkene from an alcohol with C–C bond formation; (b) linear route in three steps to prepare a nitrile from an alkene; (c) synthetic route to prepare an epoxide from a terminal alkyne, implying a “jump” from Block 1 to Block 2; two different ways from the alkene to the product are marked in red and blue; and (d) two routes to prepare amines from a carboxylic acid: the blue route requires a C–C bond forming reaction, while the red route only involves functional group transformations.

Figure 5 showcases various examples extracted from exams, which illustrate different types of solved synthetic problems with increasing levels of complexity. Figure 5a shows an example of a simple, linear reaction around the core central triangle. The starting material is an alcohol and is converted to alkene with the formation of a carbon-carbon bond. In Figure 5b, we present an example of a linear synthetic route implying two steps: the starting material is an alkene and is converted to a nitrile. Figure 5c illustrates an instance of a more complex synthetic route: the student is asked to synthesize an epoxide from a terminal alkyne with the formation of a C–C bond. After adding the remaining carbons, we observe that the alkyne formed is also present in block 2. Using the dashed arrow, we can “jump” to the green zone and continue the route from the alkyne to the epoxide. Two different paths to arrive at the final product from the alkene are marked in blue and red. Finally, Figure 5d shows two possible routes to access an amine from a carboxylic acid. The first route requires a C–C bond formation, while no C–C bond is formed in the second.

### Evaluation of the Synthetic Map Tool

We conducted a voluntary survey to gain insights into how students utilized the Synthetic Map and its impact on learning. The list of questions can be found in the Supporting Information (S3).

We first asked the students for their general opinion about the Organic Chemistry II course (Figure S1). Students found the subject relevant to their education in Pharmacy, rating it 7.9 out of 10. Their motivation for the course was rated 7/10. However, students considered the course rather difficult, with a rating of 7.3/10. When asked about the most challenging aspects of the subject, students pointed out the overwhelming amount of information and reactions to memorize (32%) as the primary difficulty. They also found it conceptual in nature (20%), difficult to understand reaction mechanisms (20%), and challenging to differentiate between different functional groups (8%). 24% of students found all of these issues problematic.

**Figure 6:**
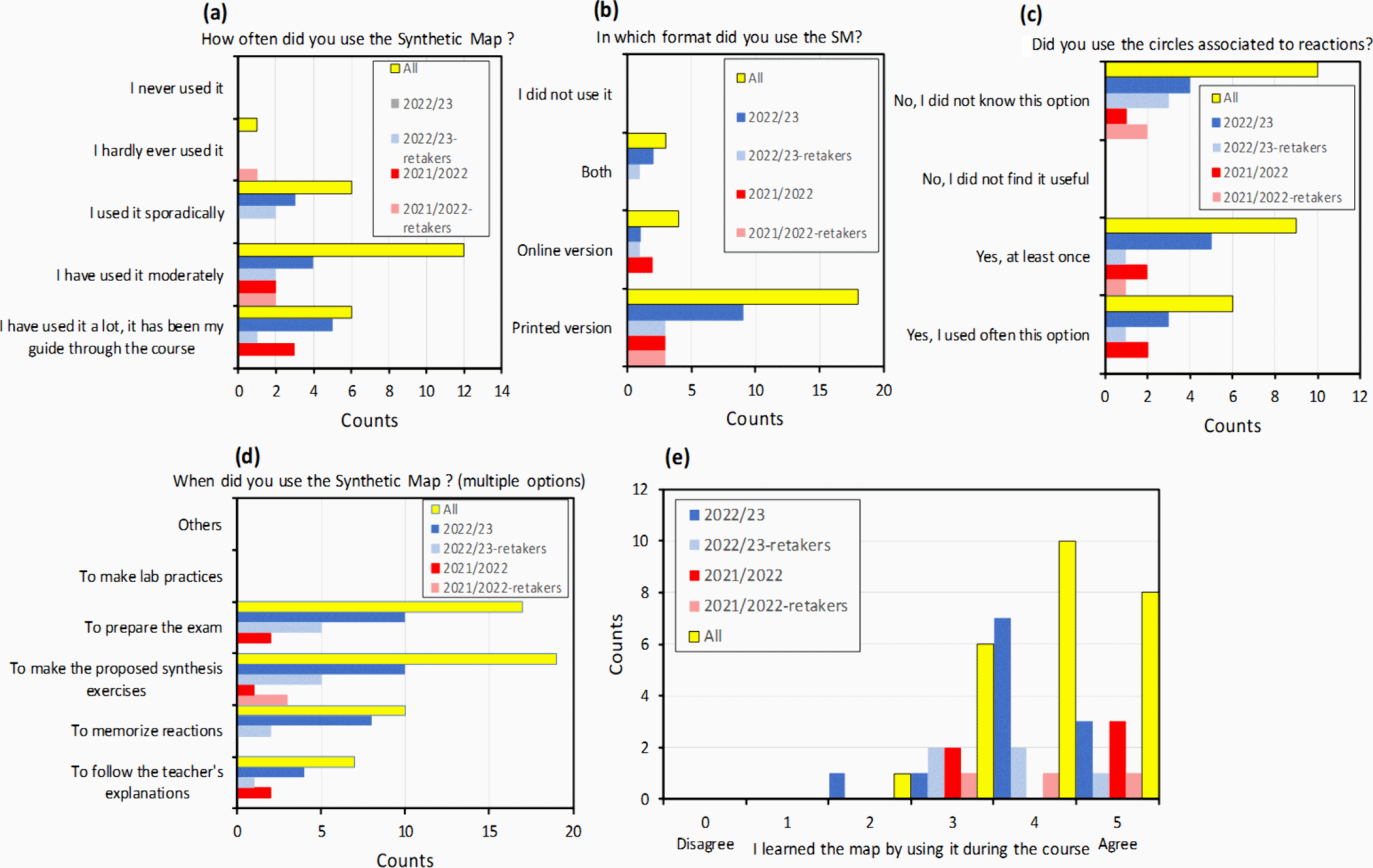
Results of the survey: questions inquiring about the use of the Synthetic Map: (a) frequency of use; (b) online or printed version of the map; (c) use of the “blue circles” in the map associated with the reactions; (d) moments of use; and (e) learning of the Synthetic Map during the course.

The subsequent survey questions aimed to gather information about utilizing the Synthetic Map (Figure 6). In terms of frequency of use, 48% of students reported using the map extensively (“*it was my guide through the course*”) or moderately (25%). The majority of students utilized the printed version of the Synthetic Map (80%), with a smaller percentage using the online version (13%) and only a few employing both (7%). 39% of students reported frequently using the numbered links directing to additional information on the reactions, and 44% had used it at least once.

We also inquired about the purposes and moments at which students employed the Synthetic Map. The primary usage was for completing synthesis exercises (36%) and preparing for exams (32%), but also for memorizing reactions (19%) and following the teacher’s explanations during class (13%). Most students agreed they learned how to use the map by practice (Figure 6e).

**Figure 7:**
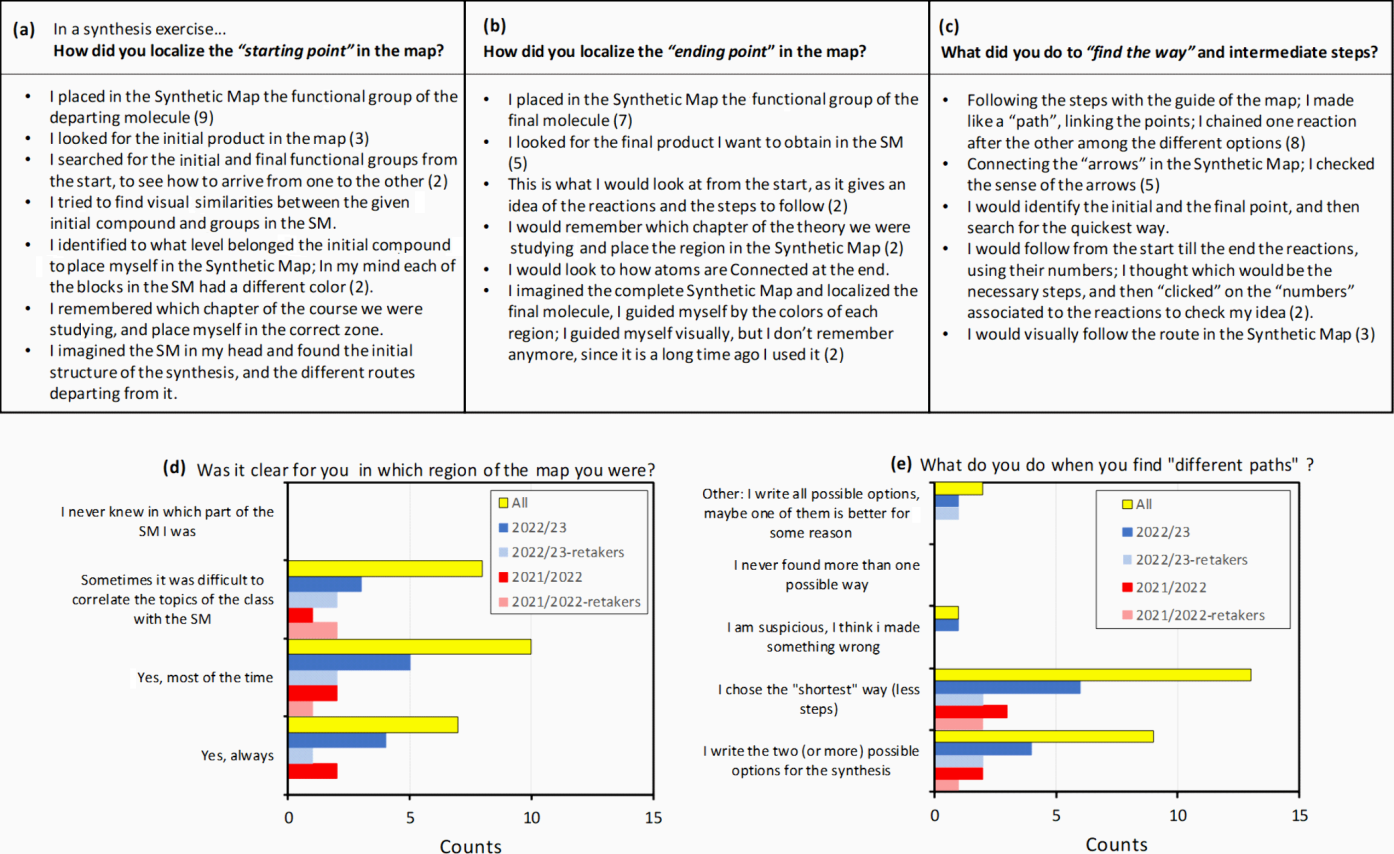
Results of the survey, regarding usage of the Synthetic Map as a “roadmap”.

To gain a deeper understanding of how students utilized the Synthetic Map in synthesis exercises and whether it was used as a “cognitive map,” we asked them to describe in their own words how they located the starting and end points of the synthesis, as well as the intermediate steps. Student responses (summarized in Figure 7a–c) revealed consistent patterns. To find the “starting point,” students looked for the functional group of the departing molecule or the initial product on the map. Some also mentioned conducting retrosynthesis by identifying the final molecule from the start. To locate the “final point,” students searched for the functional group of the final molecule or product on the map. Notably, many mentally visualized the map and placed the departing and final molecules in the color-coded blocks stored in their memory. Some relied on class theory to position themselves correctly on the map. They followed the steps and connected the arrows in the map, visually tracing the route from the initial to the final point and choosing the shortest way.

We also investigated whether the possibility of finding multiple synthesis pathways in the Synthetic Map posed a challenge for students, as earlier described for “dualistic thinkers”.^13^ On the contrary, most students (52%) reported that when they found several possible routes, they would use the map to choose the way with fewer steps. 36% expressed they would write down the different possible synthetic routes for further consideration.

**Figure 8:**
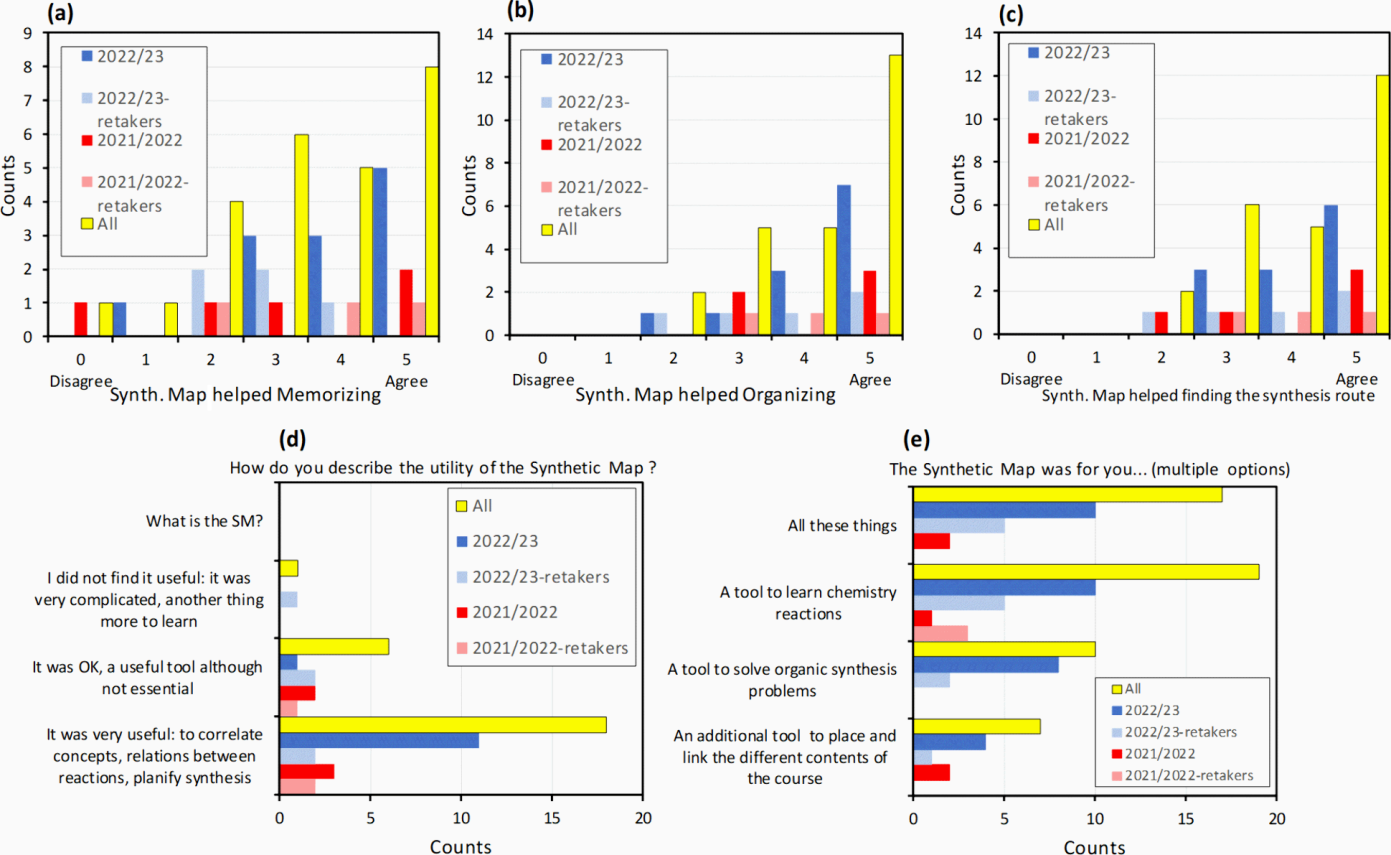
Results of the survey, regarding the student’s perception of the utility of the Synthetic Map and its benefits for learning synthesis in Organic Chemistry.

The next pool of questions was oriented to investigate whether the map was indeed helpful in addressing the challenges identified in learning synthesis reactions in Organic Chemistry. Results are summarized in Figure 8. Most students considered that the map aided them in memorizing the chemical reactions, with a level of agreement of 3.5 out of 5. Additionally, they expressed that the map helped them organize and classify the different chemical reactions in their minds (rating 4.2/5). Moreover, the map was found beneficial in assisting students in finding the route and steps for conducting an Organic Chemistry synthesis (4.1/5).

A significant proportion of students (78%) considered the Synthetic Map highly useful in correlating concepts, establishing connections between reactions, and planning synthesis. When further inquired about the utility of the map tool, students indicated that it primarily served them in learning chemistry reactions (36%), solving synthesis problems (32%), and placing and linking the different course contents (19%). 16% of students found the map tool beneficial for all these purposes.

Furthermore, to assess the map’s impact compared to other educational resources, we asked students for feedback about the most important factors to understanding Organic Chemistry II, see Figure S2a. Among the various options provided, students identified the most significant elements: solving exercises and questions (22%), attending lessons (21%), and using the Synthetic Map (20%), ranking higher than other options such as memorizing reactions (6%), attending a private academy (3%), studying with online videos (2%), or using other alternative materials (1%). Notably, for retakers, the benefit of using the Synthetic Map to comprehend the subject matter was considered more important than simply revisiting the entire course.

Finally, retaker students of 2021/2022 were asked about the principal improvements in the course compared to the previous year, when the Synthetic Map was not implemented yet (Figure S2b). All students highlighted the improvement of the supporting educational material, including the slides referencing the map. Additionally, two-thirds pointed out the benefits of the map to link all the course concepts, the practical questions to revise the theoretical concepts, the teacher’s way of explaining concepts differently, and the summary provided at the end of every class—these factors were considered more relevant than simply covering the subject again.

## Conclusions

In summary, we have developed the “Synthetic Map”, a visual cognitive map inspired by the principles of Artificial Neural Networks (ANN). It connects all the most important functional groups through their corresponding reactions. The designed Synthetic Map recreates the neural network of an expert, allowing students to gradually build their own ″mental map″ through active engagement with the course material. This approach fosters a deeper understanding of the subject and promotes acquiring, retaining, and applying new conceptual knowledge. It also encourages students to seek explicit conceptual connections between new concepts and those they already have.

We have demonstrated the educational benefits of the “Synthetic Map” by testing the tool in an Organic Chemistry course for Pharmacy students over two years. Complete map integration into the course structure was used to illustrate interrelated concepts and reactions and anchor them to existing ideas. Specific exercises were designed to help students internalize the map structure and reactions and develop their synthesis abilities. The results of our survey reveal how students used the Synthetic Map similar to an underground map to solve synthetic exercises, with the majority of students considering it a very useful tool for mastering reactions, planning synthesis, and solving complex synthetic problems with confidence. Thus, the ANN-inspired Synthetic Map represents an enriching novel tool for the teaching and learning of organic synthesis.

## Supporting Information Available

S1. Basic reactions included in the Synthetic Map. S2. Exercises to teach Organic Chemistry synthesis using the Synthetic Map. S3. Survey questions. S4. Results of the survey (PDF)
