# Supplementary material for: “Synthetic Map”: A Graphic Organizer Inspired by Artificial Neural Network Paradigms for Learning Organic Synthesis
Source: J Chem Educ. 2024 Sep 9;101(10):4256–67. doi: 10.1021/acs.jchemed.4c00592 (PMC11466318; doi:10.1021/acs.jchemed.4c00592)

Supplementary Information

"Synthetic Map": A Graphic Organizer Inspired by Artificial Neural Network Paradigms for Learning Organic Synthesis

Carlos Luque-Corredera[a], Elena Bartolomé[b]* and Ben Bradshaw[c]*

a. *Escola universitaria Salesiana de Sarrià (EUSS). Pg. Sant Joan Bosco, 74, 08017 Barcelona; email: cluque@euss.es*

b. *Institut de Ciència de Materials de Barcelona (ICMAB), Campus UAB, Bellaterra, email: ebartolome@icmab.es*

c. *Unitat Química Orgànica, Department de Química Farmacèutica de Facultat de Farmàcia, Universitat de Barcelona.* benbradshaw@ub.edu

**Corresponding authors*

S1. Basic reactions included in the Synthetic Map

S2. Exercises to teach Organic Chemistry synthesis using the Synthetic Map

S3. Survey questions

S4. Results of survey

S5. Instructions for customization of the synthetic map

S1. Basic reactions included in the Synthetic Map

Reaction Number	*Type of reaction*	*Reagents/Conditions*
	**Block 1** "*sp^3 hybridized*" (C-X)	
1	Radical Chlorination of alkanes	Cl_2, hv or Δ
2	Radical Bromination of alkanes	Br_2, hv or Δ
3	Radical Bromination of allylic alkanes	NBS,hv or Δ
4	Benzylic bromination	NBS,hv or Δ
5	Grignard formation	Mg
6	Formation of alcohols (S_N2)	NaOH
7	Williamson Ether Synthesis (S_N2)	RONa, RX
8	Formation of esters	(S_N2): $NaCO_2R$; (S_N1): RCO_2H
9	Formation of thiols (S_N2)	NaSH
10	Formation of thioethers (S_N2)	NaSR
11	Formation of trialkylsulfonium salts (S_N2)	RSR
12	Formation of phosphonium salts (S_N2)	PR_3
13	Interconversion of halogens (S_N2)- (Finkelstein)	NaI
14	Formation of amines (S_N2)	Excess NH_3
15	Alkylation of tertiary amines (S_N2)	RX
16	Formation of nitriles (S_N2)	NaCN
17	Alkylation of acetylide ions (S_N2)	Acetylene + $NaNH_2$
18	Cleavage of ethers under acidic conditions	HBr
19	Chlorination of alcohols (S_N2)	$SOCl_2$, pyridine
20	Bromination of alcohols	(S_N2) PBr_3; (S_N1) HBr
21	Alcohol to leaving group	TsCl/MsCl, base
22	Formation of epoxides via halohydrins	NaOH, H_2O
23	Opening epoxides with Nu under basic conditions	(Nu = RNH_2, RONa, NaCN, NaSH, RMgBr, $LiAlH_4$)
24	Opening epoxides with Nu under acidic conditions	H_2SO_4, (Nu = H_2O, ROH)
25	Elimination [E1] of 2° or 3° alkyl halides	H_2O
26	Elimination [E2] with impeded base	*t*-BuOK
27	Elimination [E2] of 1→3° alkyl halides	NaOMe, MeOH
28	Elimination [E1] of ROH (→*Z*)	H_2SO_4 & H_2O.
29	Conversion of alkyl halide to alkane (S_N2)	$LiAlH_4$
30	Conversion of Grignard to alkane	H_2O
	**Block 2** "*sp^2 hybridized*" (C-C)	
31	Addition of HX to alkenes	HCl or HBr
32	Radical addition of HBr	HBr, ROOR
33	Hydration of alkenes	H_2O & dil. H_2SO_4
34	Hydroboration	(i) BH_3, (ii) H_2O_2, NaOH
35	Addition of halogen	Br_2
36	Dihydroxylation of alkenes	OsO_4
37	Formation of halohydrins	Br_2, H_2O
38	Hydrogenation of alkenes	Pd/C, H_2
39	Hydrogenation of alkenes	Pd/C, H_2
40	Epoxidation	mcpba
41	Reductive ozonolysis	O_3, then Zn, AcOH
42	Convert 1,2 dihaloalkanes to alkynes	2x $NaNH_2$
43	Ring-opening of epoxides with acetylide ions	Alkyne + $NaNH_2$ then add to epoxide
44	Hydrogenation of alkynes	Pt/H_2
45	Lindlar Reduction to Z alkenes	Lindlar catalyst, H_2
46	Reduction of alkynes to *E* alkenes	Na/NH_3
47	Hydration of alkynes	$HgSO_4$, H_2O, H^+
48	Hydroboration of alkynes	(i) R_2BH, (ii) H_2O_2, NaOH
49	Ozonolysis of alkynes	(i) O_3 (ii) Zn, H_2O
50	Diels-Alder Reaction	Δ
51	Aromatic Chlorination	$Cl_2/AlCl_3$
52	Aromatic Bromination	$Br_2/FeBr_3$
53	Aromatic Iodination	$I_2/CuCl_2$
54	Aromatic Nitration	HNO_3/H_2SO_4
55	Friedel-Crafts Alkylation	RX/AlX_3
56	Friedel-Crafts Acylation	Acyl chloride or anhydride/AlX_3
57	Clemmensen Reduction	Zn(Hg), HCl
58	Benzylic Oxidation	$KMNO_4$, NaOH or $Na_2Cr_2O_7$
59	Aromatic Sulfonation	Fuming H_2SO_4

Reaction Number	*Type of reaction*	*Reagents/Conditions*
60	Aromatic Desulfonation	dilute H_2SO_4
61	Deprotonation of benzensulfonic acids	NaOH
62	Synthesis of Benzensulfonyls	PCl_5
63	Synthesis of sulfonamides	Amine
64	Nitro reduction	Zn(Hg)/HCl, or Fe/HCl or Pd/C, H_2
65	Formation of diazo compounds	$NaNO_2$/HCl
66	Sandemeyer Reaction	CuCl
67	Sandemeyer Reaction	CuBr
68	Sandemeyer Reaction	KI
69	Sandemeyer Reaction	CuCN
70	Shiemann Reaction	HBF_4 (X = F)
71	Hydrolysis of diazonium salts	H^+/H_2O, Δ
72	Diazo coupling	phenols or anilines
73	Nucleophilic Aromatic Substitution	Nu = H_2O, RNH_2, ROH, RSH
74	Fusion with NaOH	NaOH 350°C
75	Formation of anilines	PhCl, $NaNH_2$, NH_3
76	Formation of acetanilides	acid chloride or anhydride, pyridine
77	Carboxylation	CO_2 then H_3O^+
78	Metalation (Grignard formation)	Mg
	**Block 3** "*sp*^{2} *hybridized*" (C-O)	
79	PDC Oxidation	pyH-Cr_2O_7 (PDC)
80	PCC Oxidation	pyH-CrO_3Cl
81	Potassium Dichromate Oxidation	$K_2Cr_2O_7$
82	Jones Oxidation	CrO_3, H_2SO_4, acetone
83	$NaBH_4$ reduction	$NaBH_4$
84	$LiAlH_4$ reduction of carbonyls	$LiAlH_4$
85	Formation of acetals	ROH or $HOCH_2CH_2OH$/cat H^+
86	Deprotection of acetals	H_2O/H^+
87	Formation of thioacetals	$HSCH_2CH_2SH/ZnCl_2$
88	Hydrolysis of thioacetals	H_2O, $HgCl_2$, $CaCO_3$
89	Desulfurization of thioacetals to alkanes	Raney Ni/H_2
90	Imine formation	R^1NH_2 (amine 1°)
91	Enamine formation	R^1R^2NH (amine 2°)
92	Hydrolysis of imines and enamines	H_3O^+
93	Reductive amination to primary amine	NH_2OH, H_2, Ni
94	Reductive amination	$NaCNBH_3$
95	Wolff-Kishner reduction	NH_2NH_2/Δ
96	Addition of Grignards to aldehydes & ketones	RMgBr
97	Addition of Grignards to CO_2	(Grignard + CO_2)
98	Wittig Reaction	XPh_3PR, base (BuLi or NaH)
99	Oxidation of aldehydes to carboxylic acids	Ag_2O
100	Addition of organolithium to carboxylic acids	RLi (2 eq.)
101	Preparation of acid chlorides	$SOCl_2$
102	Hydrolysis of acid chlorides	H_2O
103	Esterification of acid chlorides	ROH, pyridine
104	Preparation of amides from acid chlorides	R_2NH
105	Double addition of organometallics to acid chlorides, anhydrides, or esters to give alcohols	RMgBr (2 equiv.)
106	$LiAl(OR)_3H$ acid chloride to aldehydes	$LiAl(OR)_3H$
107	Acid chloride to ketone	R_2CuLi
108	Preparation of anhydrides	acid chloride + RCOOH
109	Hydrolysis of anhydrides	H_2O
110	Esterification of Anhydrides	ROH, pyridine
111	Preparation of amides from anhydrides	R_2NH, base
112	Fischer Esterification	ROH, cat H_3O^+
113	Hydrolysis of esters (Saponification)	NaOH, H_2O
114	Transesterification	R^1OH
115	Preparation of amides from carboxylic acids	NH_3; Δ
116	Hydrolysis of amides catalyzed by acid	H_2SO_4, H_2O
117	Formation of nitriles from amides	$SOCl_2$
118	Formation de cyanohydrins	NaCN, HCl
119	Hydrolysis of nitriles	H_3O^+
120	Alcoholysis of nitriles: formation of esters	ROH, H_3O^+
121	$LiAlH_4$ reduction of amides to amines	$LiAlH_4$

122	Reduction of nitriles to aldehydes	DIBALH
123	Conversion of nitriles to ketones	RMgX then H_2O
124	Reduction of nitriles to amines	$LiAlH_4$
125	Gabriel synthesis (→amines 1ª)	(i) Pthalimide, KOH, RX (ii) NH_2NH_2 or H_3O^+
126	Halogenation of enols	Br_2/AcOH
127	Hell-Volhard-Zelinsky Halogenation Reaction	Br_2, PBr_3 (ii) H_2O
128	Alkylation at the alpha position	(i) LDA (ii) RX
129	Aldol Condensation	Base (KOH) + aldehyde o ketone
130	(A) Claisen Condensation<br>(B) Intramolecular Version. Dieckmann Cyclisation	NaOH or NaOR
131	Knoevenagel Condensation	piperidine, aldehyde
132	Mannich Reaction	$(CH_3)_2NH$, CH_2=O
133	Alkylation of amides 3ª at the alpha position	$NaNH_2$, RX
134	Alkylation of nitriles	(i) NaOEt (ii) RBr
135	Nitroaldol reaction	NaOH + aldehyde or ketone
136	Alkylation of b-ketoesters	(i) NaOEt (ii) RBr
137	Decarboxylation de b-ketoesters	H_3O^+/Δ
138	Malonic ester synthesis	(i) NaOEt (ii) RBr (iii) H_3O^+/Δ
139	Michael Reaction - Conjugate addition 1,4: Nu =	NaCN, H_2O, RNH_2, R_2CuLi
140	Robinson Annulation	Base (NaOH)
141	Formation of cuprates	Grignard or Organolitium + CuI
142	Addition of cuprates to electron-deficient alkynes	R_2CuLi
143	Stork enamine alkylation	pyrrolidine, then RX, acyl chloride, or Michael acceptor, or (ii) H_3O^+

S2. Exercises to teach Organic Chemistry synthesis using the Synthetic Map

1st Type of Problem Seminar: Students learn the reactions in the order presented in the course. Then, they have to fill in the reagents.

Determine the reagents for each reaction using the Synthetic Map.

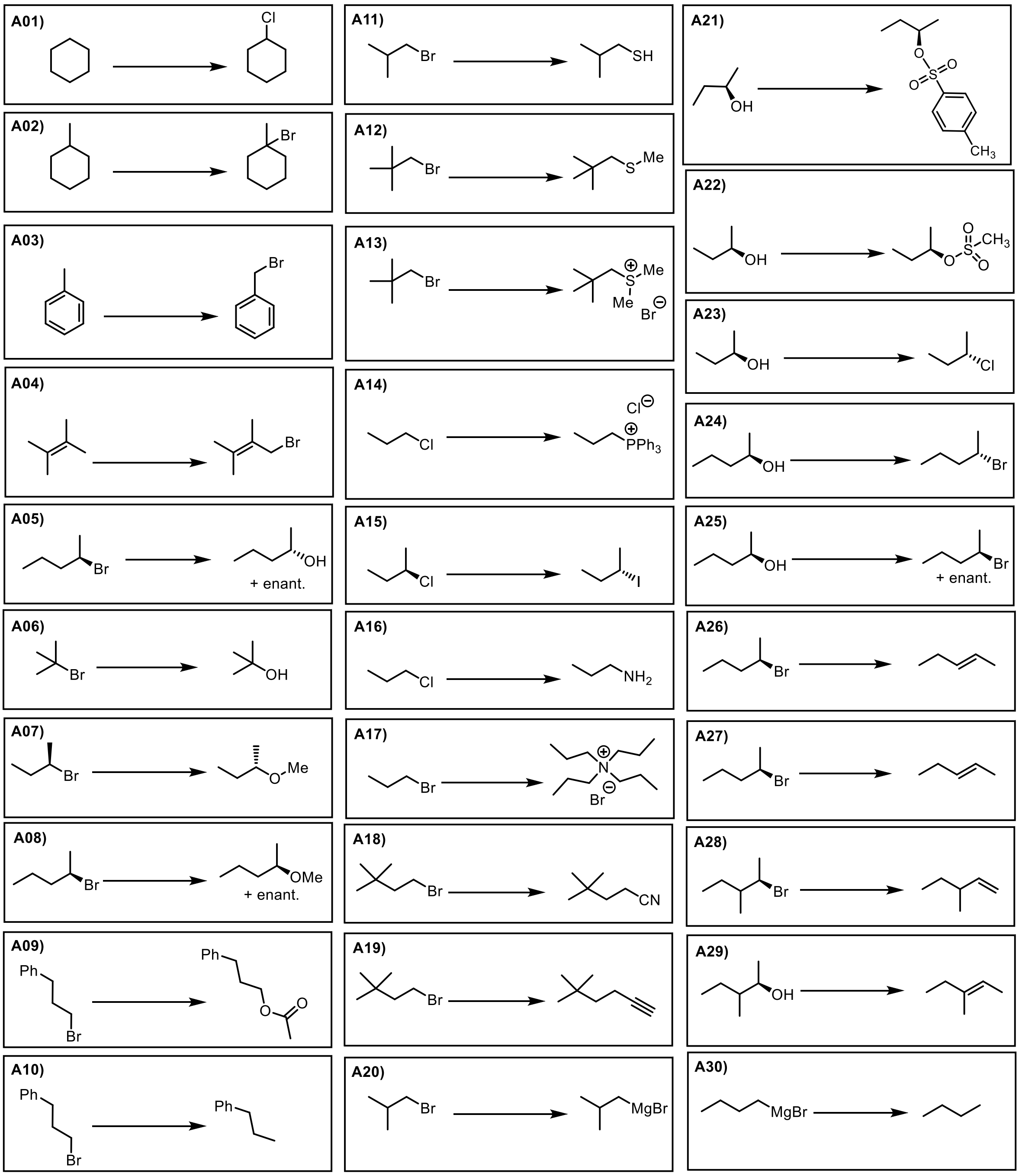

Correct Answers to 1st Type of Problem Seminar

A01)
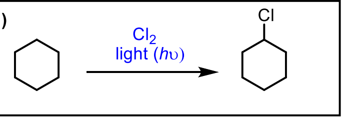

A02)
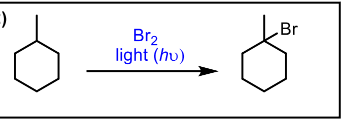

A03)
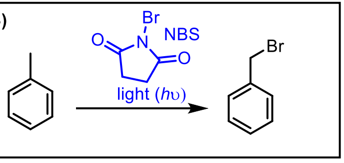

A04)
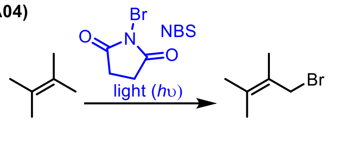

A05)
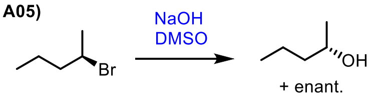

A06)
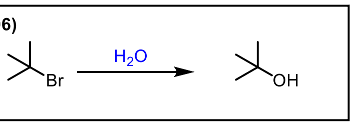

A07)
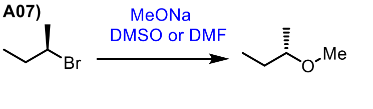

A08)
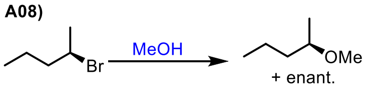

A09)
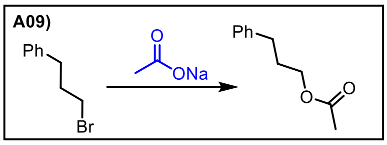

A10)
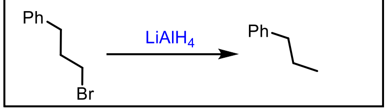

A11)
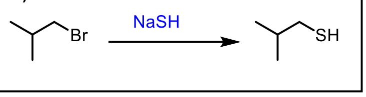

A12)
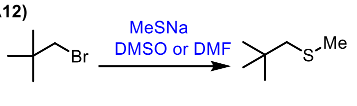

A13)
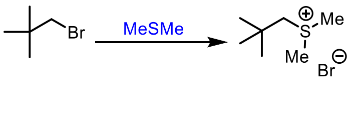

A14)
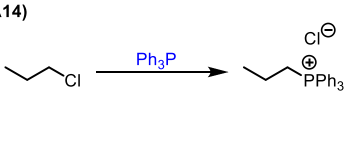

A15)
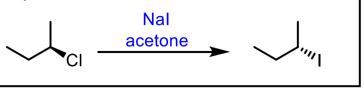

A16)
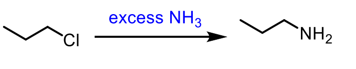

A17)
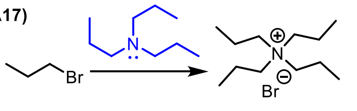

A18)
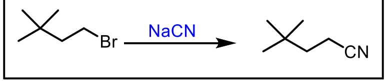

A19)
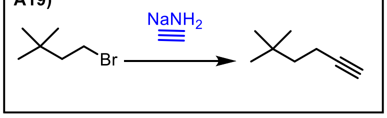

A20)
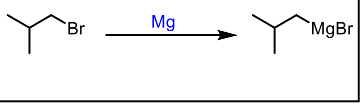

A21)
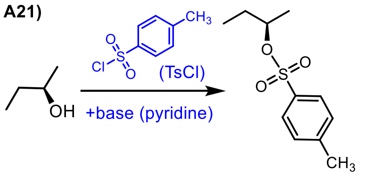

A22)
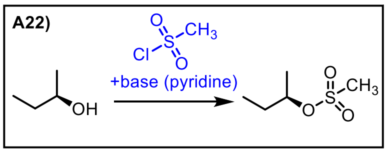

A23)
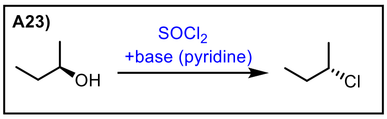

A24)
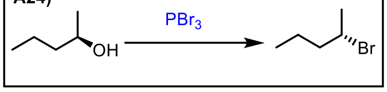

A25)
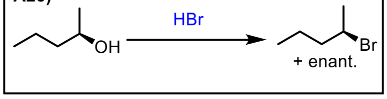

A26)
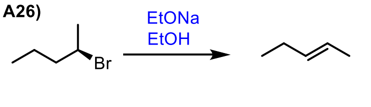

A27)
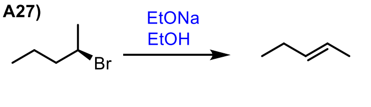

A28)
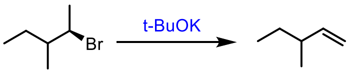

A29)
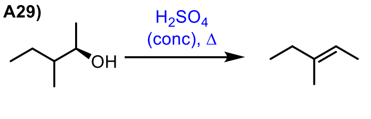

A30)
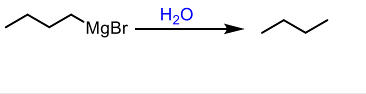

2nd Type of Problem Seminar: Here, the difficulty level is higher: The reactions are presented in random order, and now the student must determine either the starting material, reagent, or final product missing in each case.

Using the Synthetic Map determine the missing starting material, reagent or final product required in each scheme.

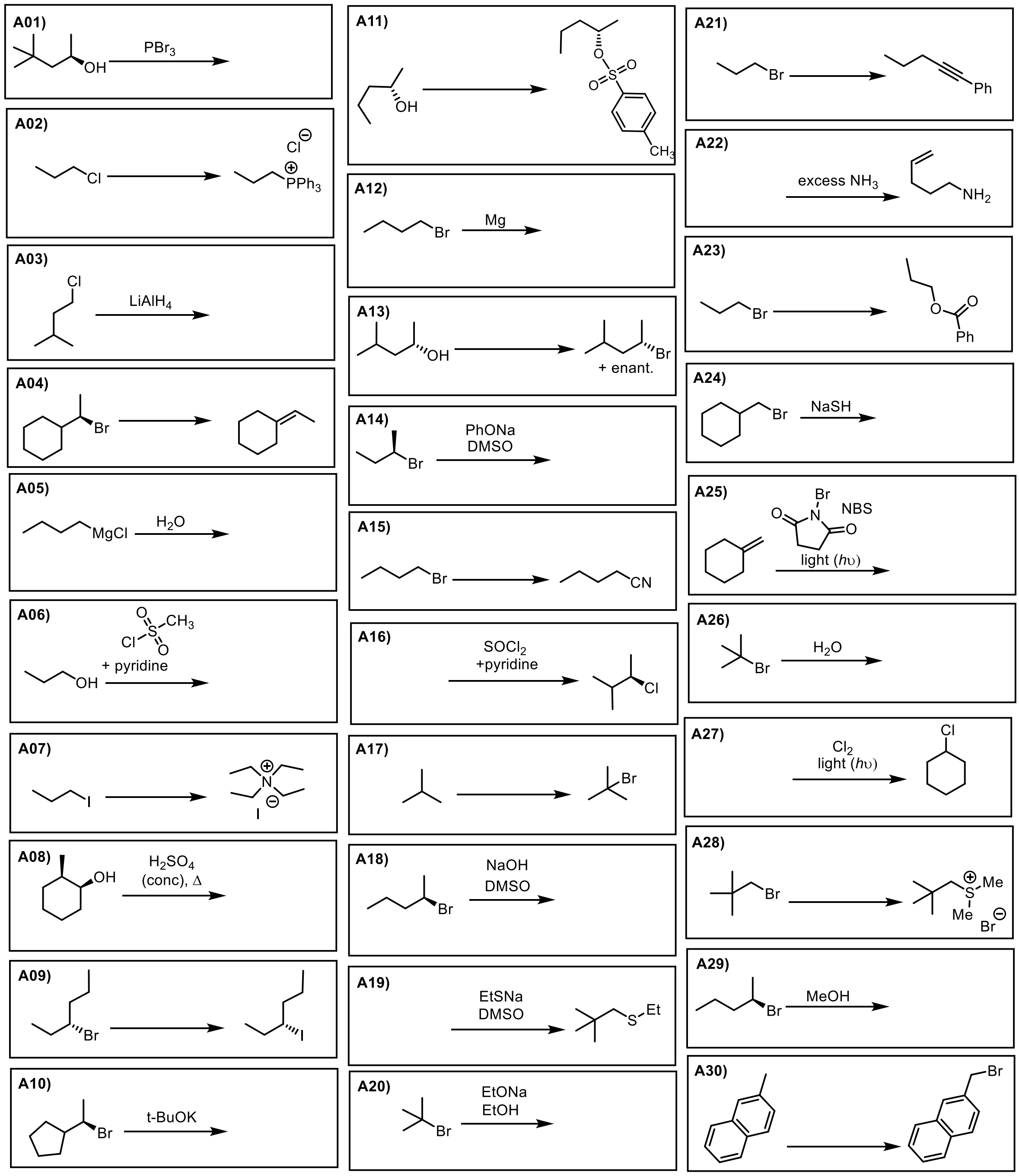

Correct Answers to 2nd Type of Problem Seminar

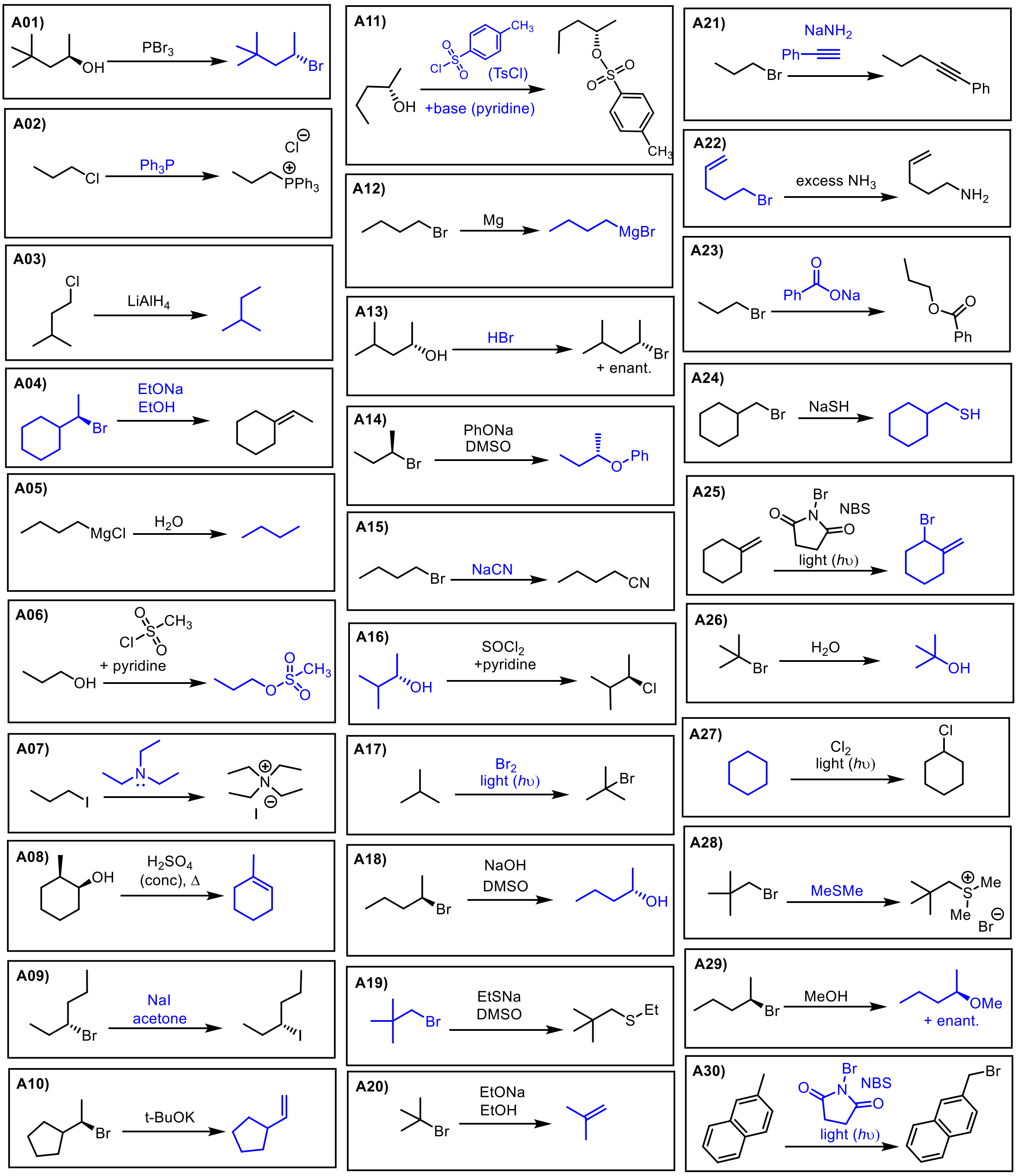

3rd Type of Problem Seminar: This exercise builds on the previous two exercises by asking the student to determine the reagent needed in each case to complete part of the synthetic map.

Add the reagents needed for the following reactions on each arrow:

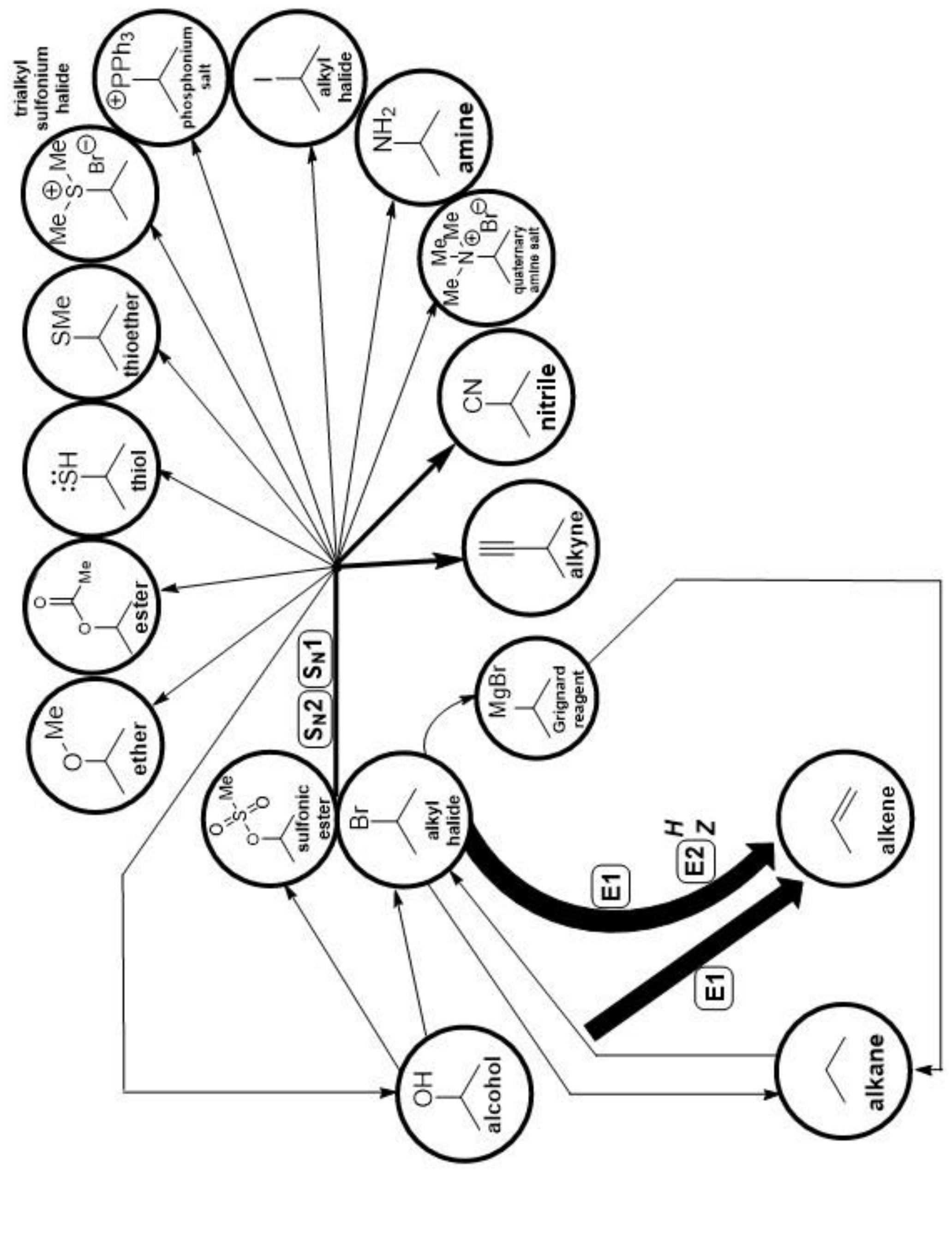

Correct Answers to 3rd Type of Problem Seminar

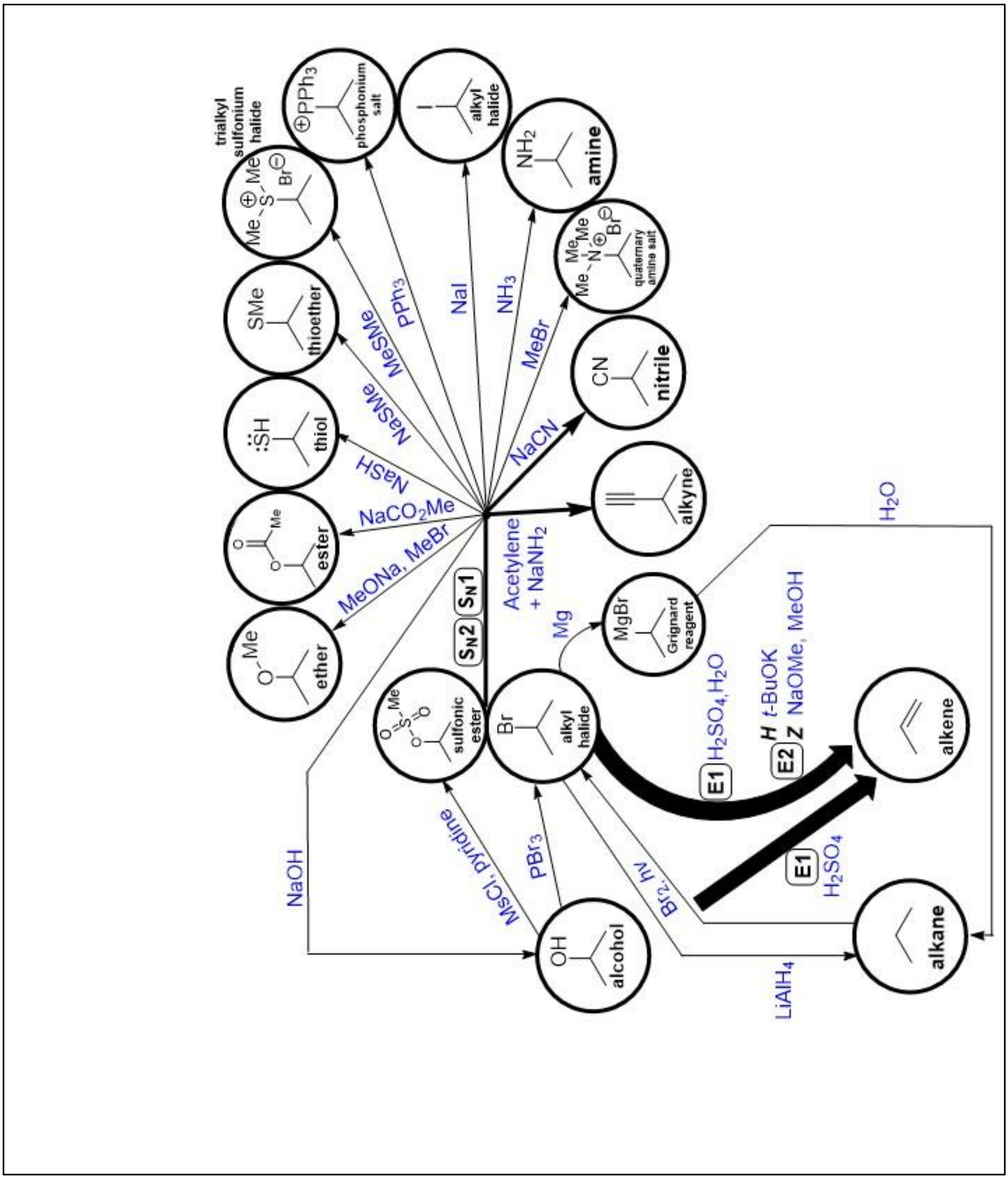

4th Type of Problem Seminar: this exercise builds on the previous three exercises by asking the student to complete part of the synthetic map from memory using only a basic outline of the map structure.

Fill in the missing functional groups and suggest suitable reagents for each transformation.

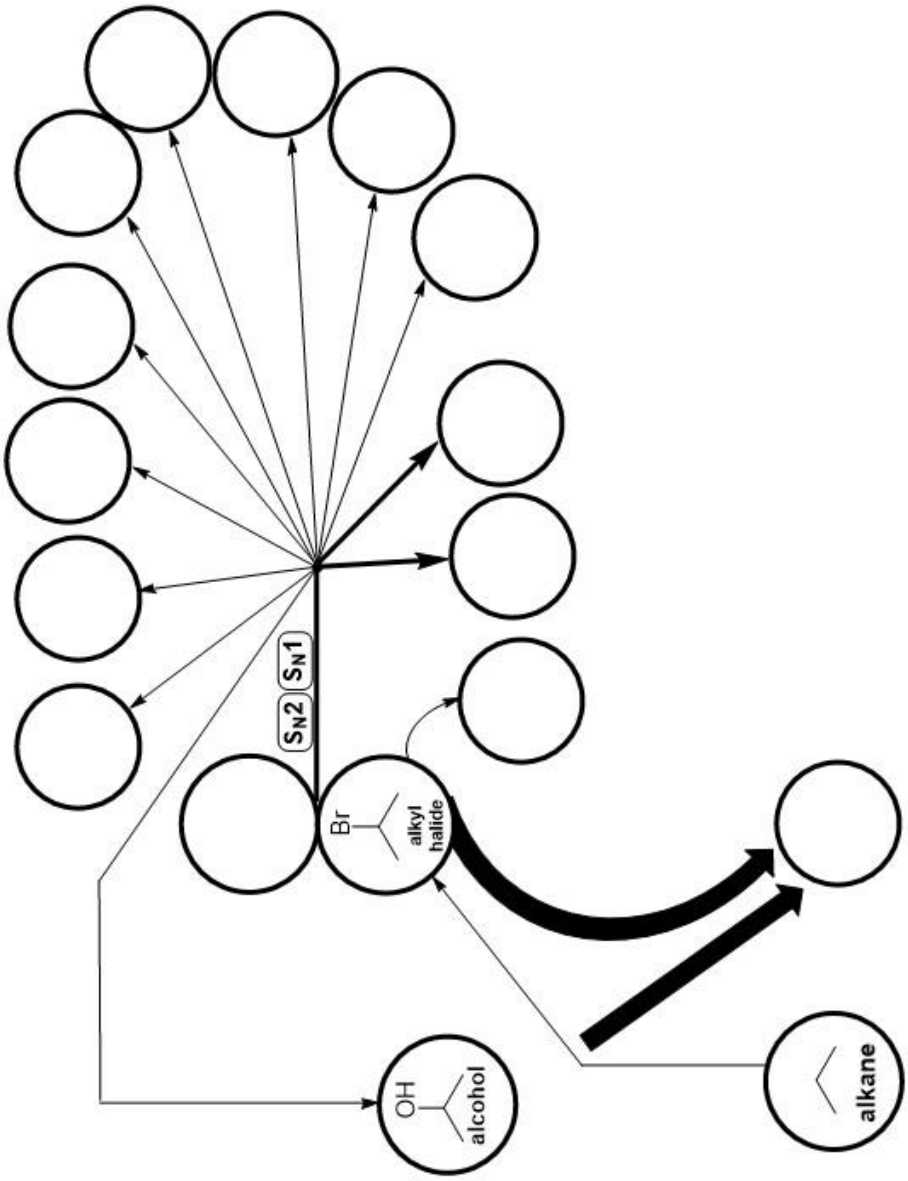

Example of a Student Answer to the 4th Type of Problem Seminar

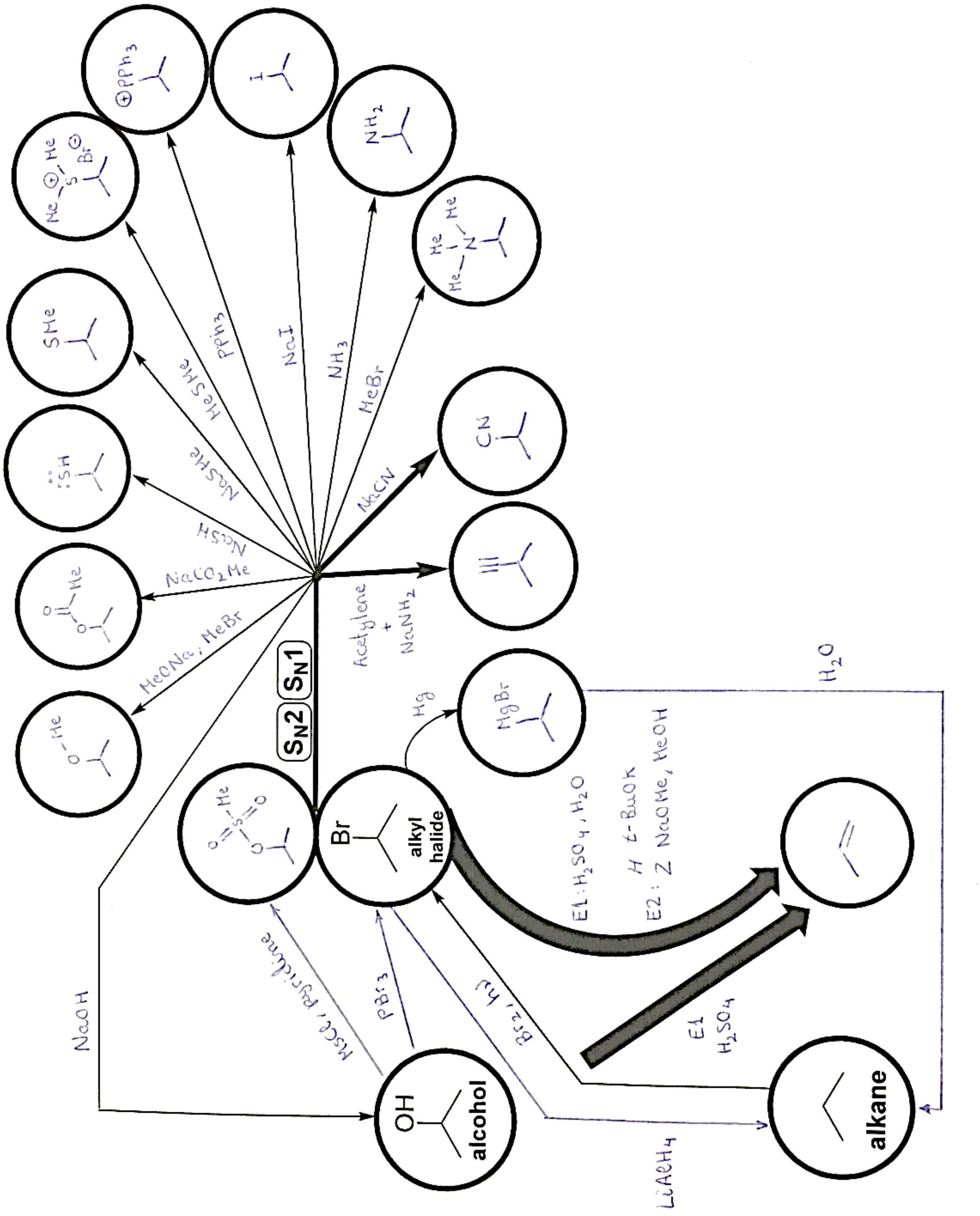

S3. Survey questions

This investigation took place within the Pharmacy degree program at the University of Barcelona (UB), involving students enrolled in the Organic Chemistry II courses for the academic years 2021/22 and 2022/23. The survey, prepared on Google Forms, was distributed to participants via email. Participation in the survey was voluntary. To adhere to ethical standards, the purpose of the survey was communicated in the survey heading, we guaranteed participants' anonymity and made it clear that their responses would not influence their grades. Twenty-five students responded to the survey, including 17 from the 2021/22 course (5 retakers) and 8 from the 2022/23 course (3 retakers).

Initial categorization of students was made through question (1). Depending on the category, the participants received an additional part of the questionnaire. These were the questions posed to each subgroup:

A) Students taking for the first time OC(II) in 2022-2023 (2nd year of Synthetic Map implementation): questions 1 to 31.
B) Students retaking OC(II) in 2022-2023 as retakers (they used the Synthetic Map two consecutive years): questions 1 to 31.
C) Students who took OC(II) in 2021-2022 only (1st year of Synthetic Map implementation): questions 1 to 31.
D) Students who took OC(II) in 2020-2021 (Synthetic Map not implemented), failed, and studied the course again in 2021-2022 using the Synthetic Map: questions 1-5, and 23 to 31.

Questionnaire:

1) In which academic year did you study Organic Chemistry (II)?

☐ This academic course 2022-2023 for the first time.
☐ Last year's course 21-22, and also this course 22-23 as a retaker.
☐ The last course 21-22 only.
☐ In the course 20-21, and also the last year's course 21-22 as a retaker.

2) **How difficult** is the subject (Organic Chemistry – II) for you?

(Very easy) 1 ☐ 2 ☐ 3 ☐ 4 ☐ 5 ☐ 6 ☐ 7 ☐ 8 ☐ 9 ☐ 10 ☐

3) What do you think is the **most difficult part of the course**, and why?

☐ There is too much information (many reactions to memorize).
☐ I don't understand the differences between functional groups and their reactivity.
☐ For the same synthesis exercise there are many valid answers.
☐ Understand the reaction mechanisms.
☐ It is a conceptual subject that above all requires understanding more than memory.
☐ All the previous answers.
☐ Others: ……………………………

4) Regardless of the difficulty, what is your **motivation/interest** in the subject?

(It doesn't motivate me at all) 1 ☐ 2 ☐ 3 ☐ 4 ☐ 5 ☐ 6 ☐ 7 ☐ 8 ☐ 9 ☐ 10 ☐ (It motivates me)

5) Evaluate the **usefulness/relevance** of Organic Chemistry II for your training in the Pharmacy university degree.

(useless/unnecessary) 1 ☐ 2 ☐ 3 ☐ 4 ☐ 5 ☐ 6 ☐ 7 ☐ 8 ☐ 9 ☐ 10 ☐ (very useful/necessary)

6) How do you rate your **experience using the "Synthetic Map"**?
☐ It has been very useful for relating concepts, relationships between reactions, planning synthesis...
☐ It's been fine, a useful tool, though not essential.
☐ I have not seen it very useful: it was very complicated, and one more thing to learn...
☐ What's the "Synthetic Map"?
☐ Others: ……………………………

7) **How often** have you used the "Synthetic Map"?
☐ I have used it a lot, it has been my guide during the course.
☐ I have used it moderately.
☐ I have used it sporadically.

☐ I have hardly ever used it.
☐ I have never used it.

8) **In what format** did you use the "Synthetic Map"?

☐ In printed format (on paper).
☐ In digital format.
☐ In both formats.
☐ I haven't used it.

9) In the digital format, have you clicked on the numbered circles to get more information about the reactions?

☐ Yes, I have used this option quite a lot.
☐ Yes, at least once.
☐ No, I didn't find it useful.
☐ No, I didn't know about this option.

10) Do you think that the "Synthetic Map" has helped you to **memorize** the different chemical reactions?

(I disagree) 1 ☐ 2 ☐ 3 ☐ 4 ☐ 5 ☐ 6 ☐ 7 ☐ 8 ☐ 9 ☐ 10 ☐ (I agree)

11) Do you think that the "Synthetic Map" has helped you **mentally classify and organize** the different chemical reactions?

(I disagree) 1 ☐ 2 ☐ 3 ☐ 4 ☐ 5 ☐ 6 ☐ 7 ☐ 8 ☐ 9 ☐ 10 ☐ (I agree)

12) Do you consider that the Synthetic Map has helped you to **find the path and the steps to follow** to carry out a chemical synthesis?

(I disagree) 1 ☐ 2 ☐ 3 ☐ 4 ☐ 5 ☐ 6 ☐ 7 ☐ 8 ☐ 9 ☐ 10 ☐ (I agree)

13) **At what point(s) have you used the "Synthetic Map**"? (several answers possible)

☐ To follow the teacher's explanations during the class.
☐ To memorize the reactions.
☐ When doing the proposed synthesis exercises.
☐ To prepare for the exam.
☐ To help me do the lab practices.
☐ Others: ……………………………

14) The Synthetic Map has been for you...

☐ An additional tool of Organic Chemistry II to locate and unite the contents of the subject.
☐ A tool for solving chemical synthesis problems.
☐ A tool to understand chemical reactions.
☐ Others: ……………………………

15) While you have studied the subject, have you been clear in which **area of the map** you were at all times?

☐ Yes, at all times.
☐ Yes, in most of the times.
☐ Sometimes it was a bit difficult to relate the topic of the class to the Map.
☐ I have never known in which region of the map I was.
☐ Others: ……………………………

16) Do you agree with this statement?: "***As I have been using the map during the classes, I have been assimilating it***".

(I disagree) 1 ☐ 2 ☐ 3 ☐ 4 ☐ 5 ☐ 6 ☐ 7 ☐ 8 ☐ 9 ☐ 10 ☐ (I agree)

17) In a synthesis exercise, how do you locate the "**starting point**" on the "Synthetic Map"? (Explain in your own words).

……

18) In a synthesis exercise, how do you locate the "**ending point**" on the "Synthetic Map"? (Explain in your own words).

…….

19) How do you find the **path and the intermediate stages between the starting point and the ending point** using the "Synthetic Map"? (Explain in your own words)

……

20) What do you do when there are **several possible paths** in the "Synthetic Map" to reach the solution of a problem?

- ☐ I write the two (or more) possible options to complete the synthesis.
- ☐ I choose the shortest path (with fewer steps).
- ☐ I'm suspicious, I think there's something I've done wrong.
- ☐ I have never come across more than one possible path.
- ☐ Others: …………………………..

21) What have been the **most important factors in understanding Organic Chemistry II**?

- ☐ Attend class.
- ☐ Do the proposed exercises and questions.
- ☐ Memorize the reactions just before the exam.
- ☐ Attend an academy or private classes.
- ☐ Study the support material for the subject (theory slides).
- ☐ Study other support material for the subject (textbooks, websites, blogs...)
- ☐ Study by watching educational videos online.
- ☐ Use the "Synthetic Map" to plan the syntheses and relate the concepts.
- ☐ Others: …………………………..

22) Do you have any suggestions to improve the "Synthetic Map"?

- ☐ Yes
- ☐ No
- ☐ Others: …………………………..

23) **Comparing** your experience of doing Organic Chemistry II **with and without the "Synthetic Map"**, what is your assessment?

- ☐ The "Synthetic Map" was very useful: to relate concepts, relationships between reactions…
- ☐ It was fine, a useful tool, though not essential.
- ☐ Study other support material for the subject (textbooks, websites, blogs...)
- ☐ I didn't find it very useful: it was very complicated, and one more thing to learn in Organic Chemistry II
- ☐ What's the "Synthetic Map"?

24) During the 21-22 academic year, **how often** did you use the "Synthetic Map"?

- ☐ I have used it a lot, it has been my guide during the course.
- ☐ I have used it moderately.
- ☐ I have used it sporadically.
- ☐ I have hardly ever used it.
- ☐ I have never used it.

25) During the 21-22 academic year, In what format did you use the "Synthetic Map"?

- ☐ In printed format (on paper).
- ☐ In digital format.
- ☐ In both formats.
- ☐ I haven't used it.

26) Comparing your experience between the academic courses 20-21 (without the Map) and 21-22 (with the Map), do you think that the "Synthetic Map" has helped you to **memorize** the different chemical reactions?

(I disagree) 1 ☐ 2 ☐ 3 ☐ 4 ☐ 5 ☐ 6 ☐ 7 ☐ 8 ☐ 9 ☐ 10 ☐ (I agree)

27) Comparing your experience between the academic courses 20-21 (without the Map) and 21-22 (with the Map), do you think that the “Synthetic Map” has helped you **mentally classify and organize** the different chemical reactions?

(I disagree) 1 ☐ 2 ☐ 3 ☐ 4 ☐ 5 ☐ 6 ☐ 7 ☐ 8 ☐ 9 ☐ 10 ☐ (I agree)

28) Comparing your experience between the academic courses 20-21 (without the Map) and 21-22 (with the Map), do you consider that the Synthetic Map has helped you to **find the path and the steps to follow** to carry out a chemical synthesis?

(I disagree) 1 ☐ 2 ☐ 3 ☐ 4 ☐ 5 ☐ 6 ☐ 7 ☐ 8 ☐ 9 ☐ 10 ☐ (I agree)

29) During the second time you studied Organic Chemistry II (academic course 21-22)**, at what point(s) have you used the “Synthetic Map**”? (several answers possible)

☐ To follow the teacher's explanations during the class.
☐ To memorize the reactions.
☐ When doing the proposed synthesis exercises.
☐ To prepare for the exam.
☐ To help me do the lab practices.
☐ Others: …………………………….

30) What have been the **most important factors in understanding Organic Chemistry II**?

☐ Attend class.
☐ Do the exercises and questions proposed.
☐ Memorize the reactions just before the exam.
☐ Attend an academy or private classes.
☐ Study the support material for the subject (theory slides).
☐ Study other support material for the subject (textbooks, websites, blogs...)
☐ Study by watching educational videos online.
☐ Use the "Synthetic Map" to plan the syntheses and relate the concepts.
☐ Repeat the subject a second time.
☐ Others: …………………………….

31) What element(s) do you consider is better, comparing both editions: the 21-22 course (with the Map) and the 20-21 course (without the Map)?

☐ Classes with well-defined objectives.
☐ Review of the previous material at the beginning of each lecture..
☐ The use of the "Synthetic Map" to link all the concepts of the course.
☐ Practical questions to review theoretical concepts.
☐ The continuous reference to the "Synthetic Map" during the lecture to encourage memorization.
☐ Summary at the end of each lecture.
☐ Study material (printed slides) containing the most important.
☐ A teacher who explains things differently.
☐ Basically, seeing the entire course a second time.
☐ Others: …………………………….

S4. Results of survey

Additional results of survey, not shown in the main text.

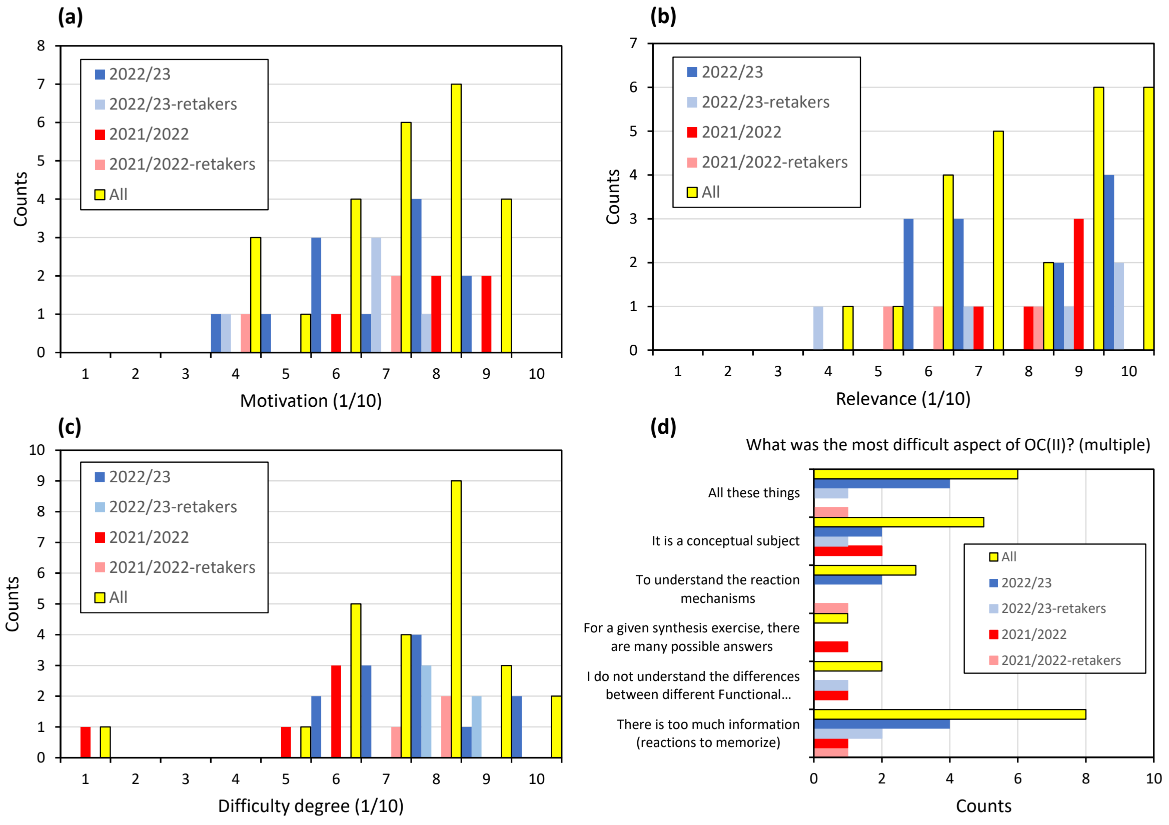

Figure S1. Results of survey: questions inquiring about (a) the Pharmacy students' motivation for "Organic Chemistry (II)" course, (b) their self-perception about the relevance of the course for their education, (c) the degree of difficulty of the course, and (d) the aspects of the subject considered most difficult.

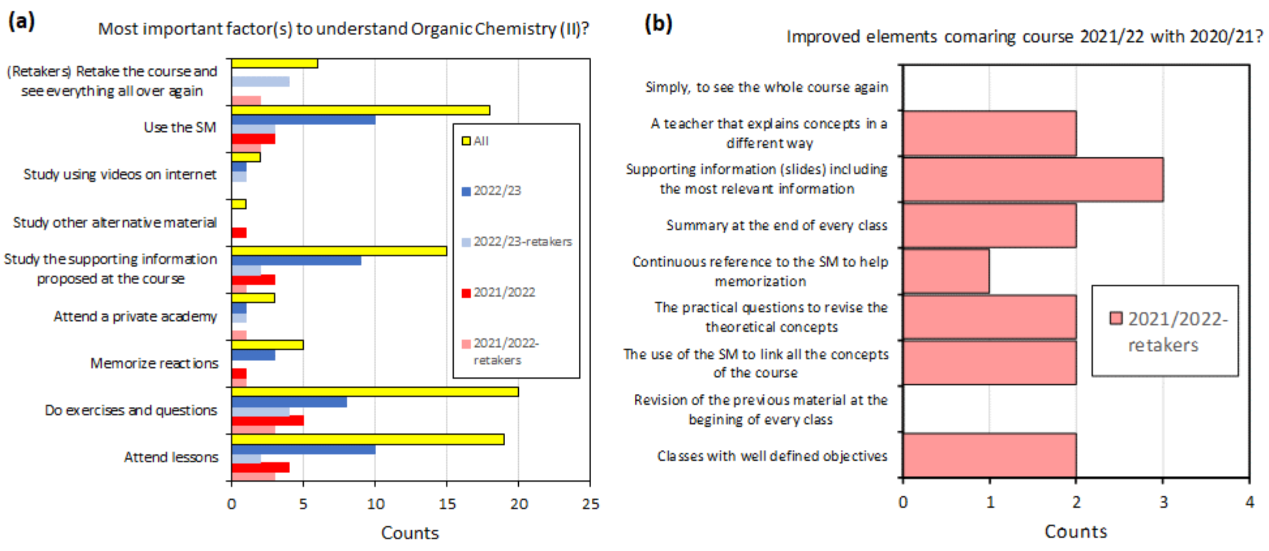

Figure S2. Results of survey: questions inquiring about the impact of the Synthetic Map.

S5. Instructions for customization of the synthetic map

In the following document, we will provide instructions on setting up your customized synthetic map.

You will require the following programs (or their equivalents):

- ChemDraw Professional
- Adobe Dreamweaver
- A web hosting service and a domain name

The basic steps to follow are the following:

1. Setup the file structure to store your customized synthetic map
2. Design your own customized synthetic map
3. Set up the main page
4. Make pages for individual reactions
5. Link the reactions to the main page of the synthetic map
6. Upload your customized synthetic map to a server

---

1. Setup the file structure to store your customized synthetic map

Set up a folder called “Synthetic map,” where you store all your files for your customized synthetic map. Inside this folder, set up a subfolder called “Reactions.”

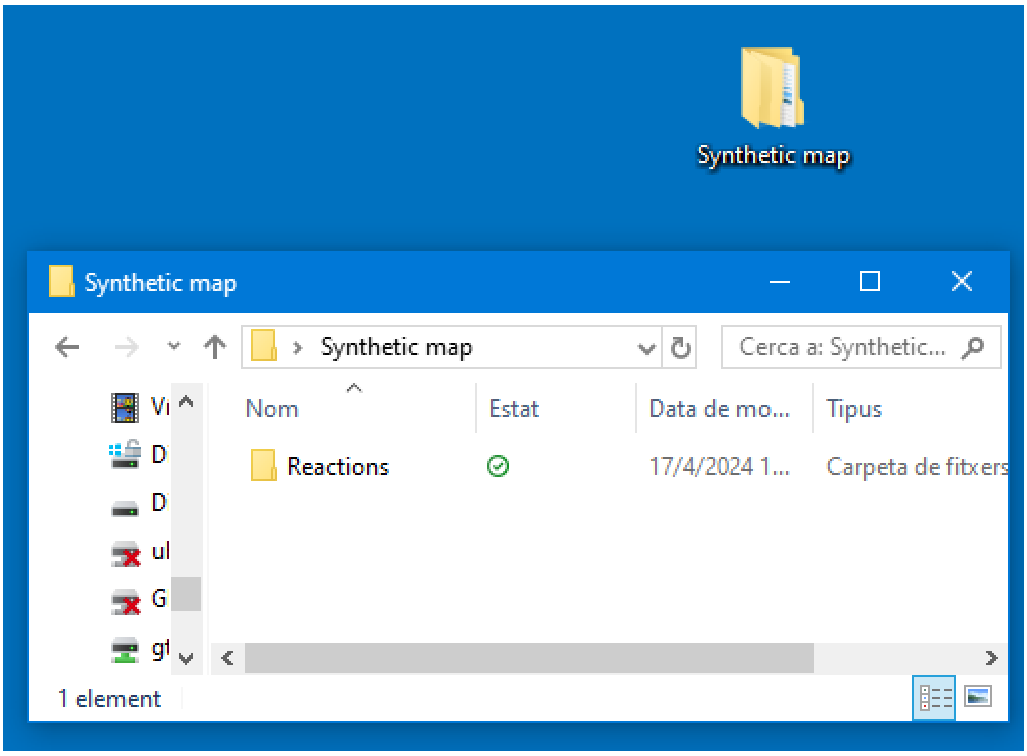

2. Design your own customized synthetic map

You can design your synthetic map from scratch using the basic design principles we outline in the "An Introduction to Synthetic Map video."

Alternatively, you can use our Chemdraw diagram (which we provide on the website as a download) as the starting point for your customized synthetic map and modify it to your needs.

For this demonstration, we will use the following very simplified version of the synthetic map:

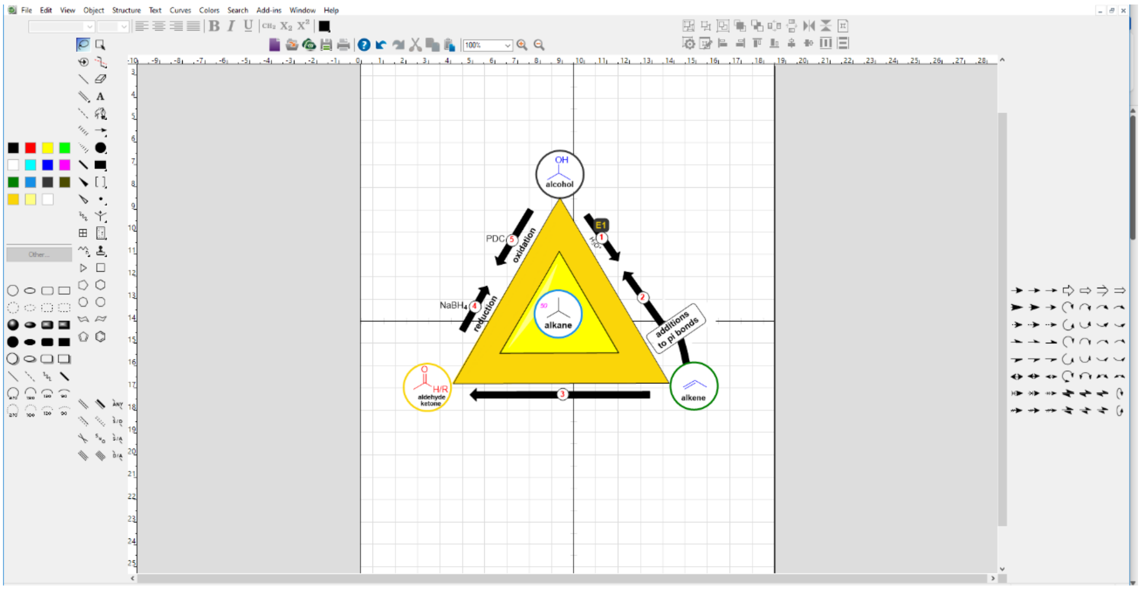

Once you have prepared your synthetic map, save the drawing in the main site folder as a chemdraw file named **Synthetic map.cdxml** and as a .gif file named **Synthetic map.gif**.

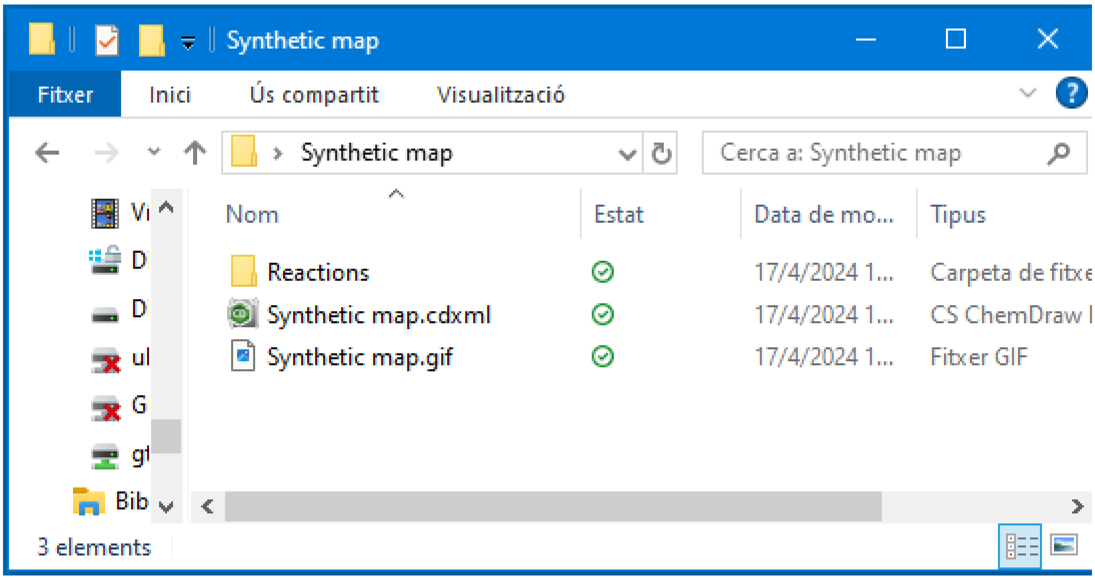

3. Set up the main page

Open Dreamweaver, go to **File**, and then **New**.

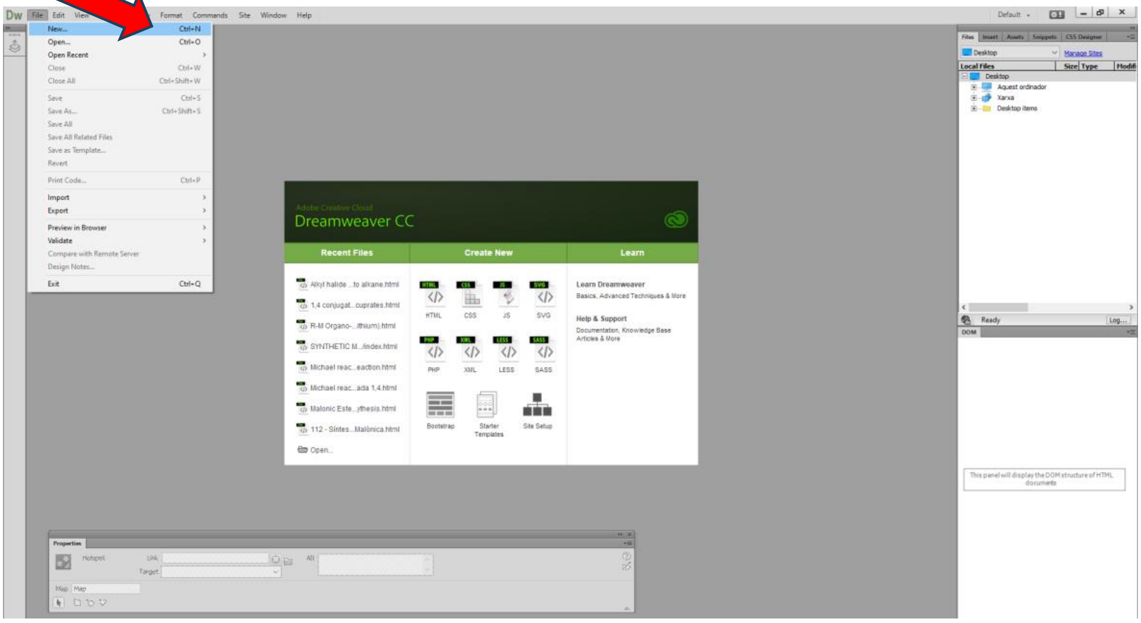

Then select the **HTML** file option and hit **Create**.

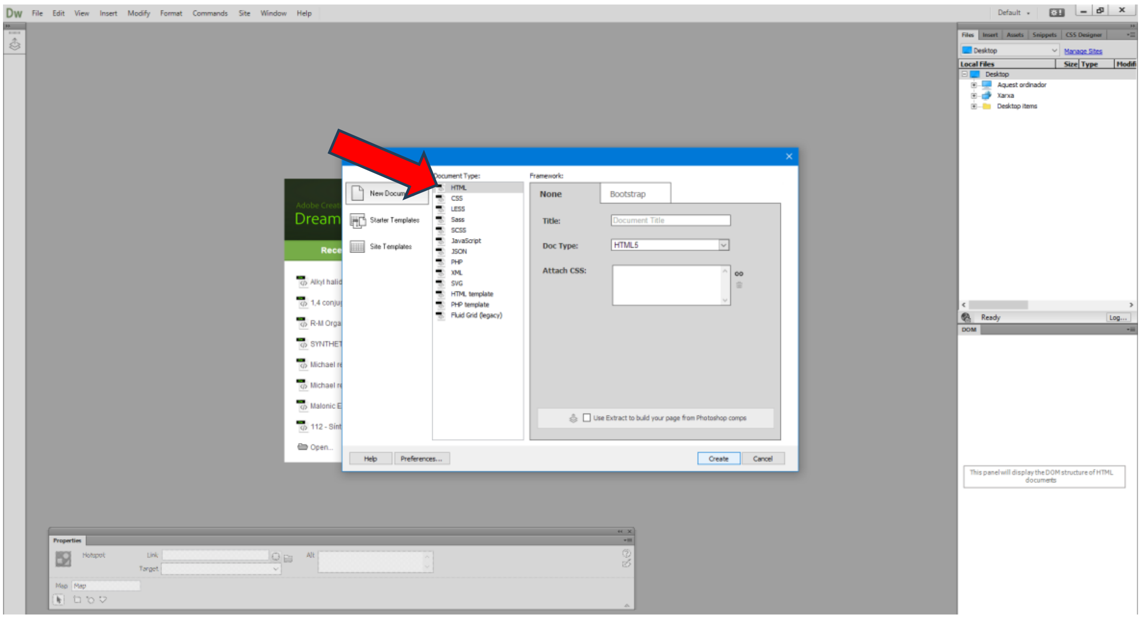

Save the page as **index.html** in the main folder.

Go to the **Insert menu** and select **table**

Settings:

- Rows: 1
- Columns: 1
- Border thickness: 0

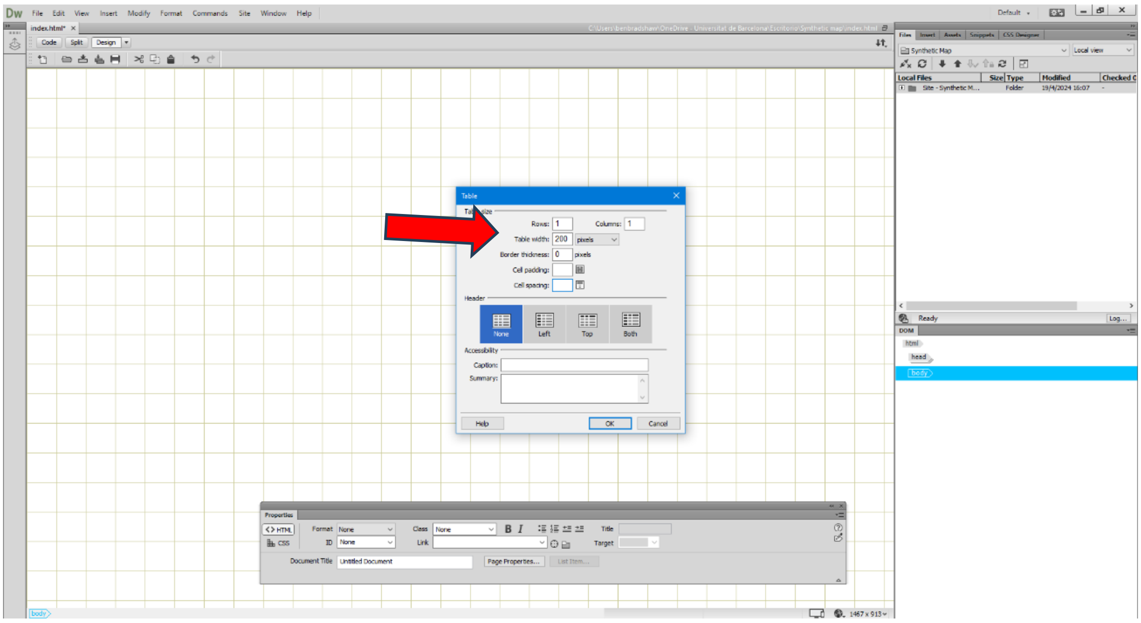

Select your table, and in the properties box, select **center.**

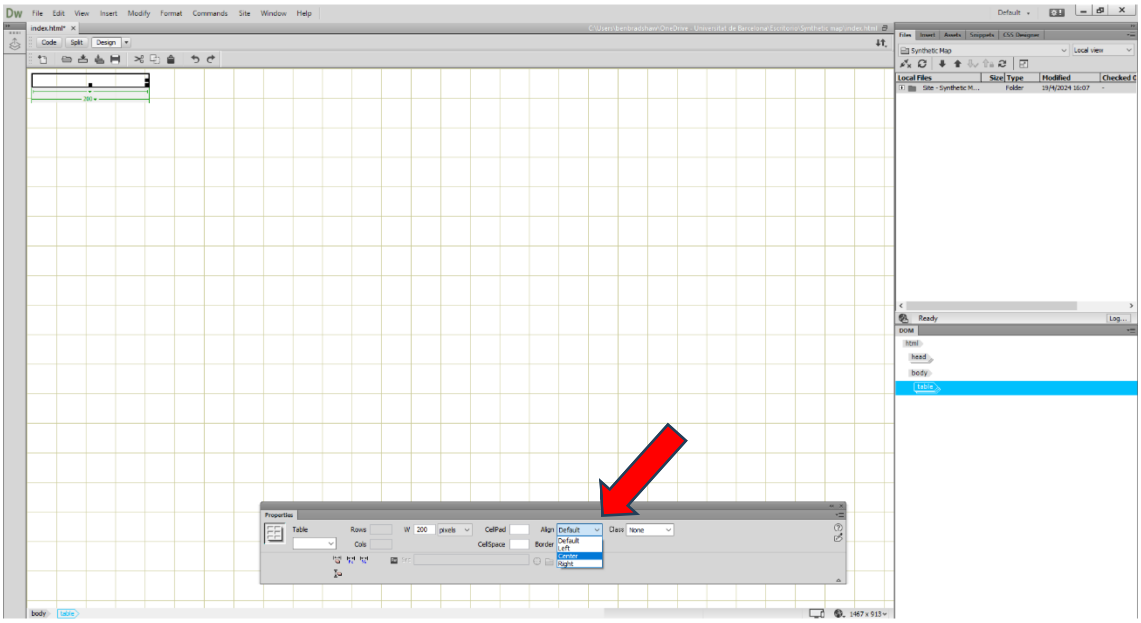

Place your cursor in your table, then go to **Insert** and select **Image**.

Select the .gif image of your synthetic map from the site folder.

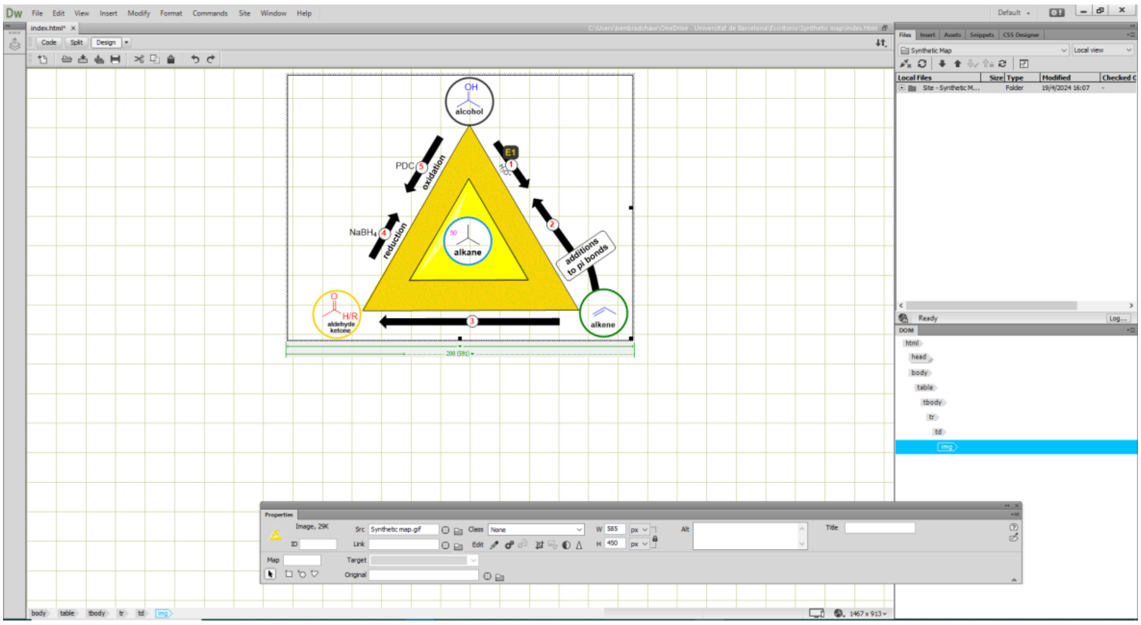

Save your work.

---

4. Make pages for individual reactions

To make your page for any reaction the process is similar to that described above.

Make a new folder for your reaction in the reactions folder set up in part 1. Use a name that describes the reaction: for example, "$NaBH_4$ reduction."

Open a new HTML file and save it in the folder you created with the same name. In Chemdraw, prepare the corresponding images required – one for the reaction (save as reaction.cdx and reaction.gif) and one for the mechanism (save as mechanism.cdx and mechanism.gif)

You should now have a folder with the following files:

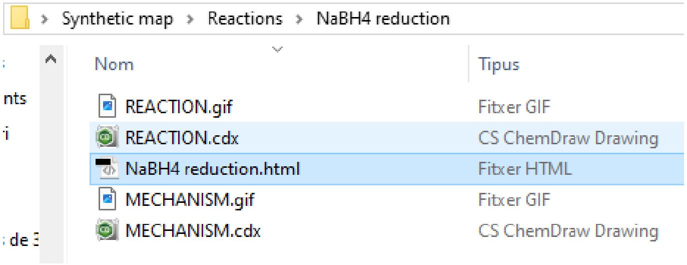

As before, insert a centered table, but this time, it should have six rows and one column.

To each row of the table, add the information required:

1. Title of the reaction
2. Reaction image (reaction.gif)
3. Information about the reaction
4. Mechanism title
5. Information about the reaction
6. Image of the mechanism (mechanism.gif)

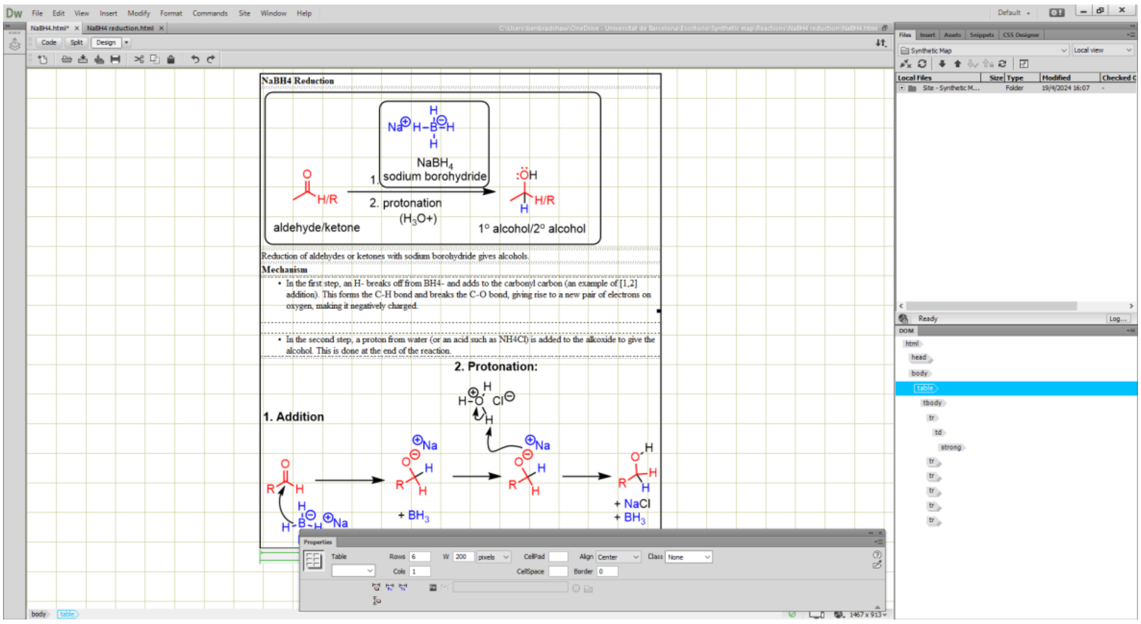

To add subindex or superindex to formulas and charges, it is necessary to do it through the code view by selecting “Split” view:

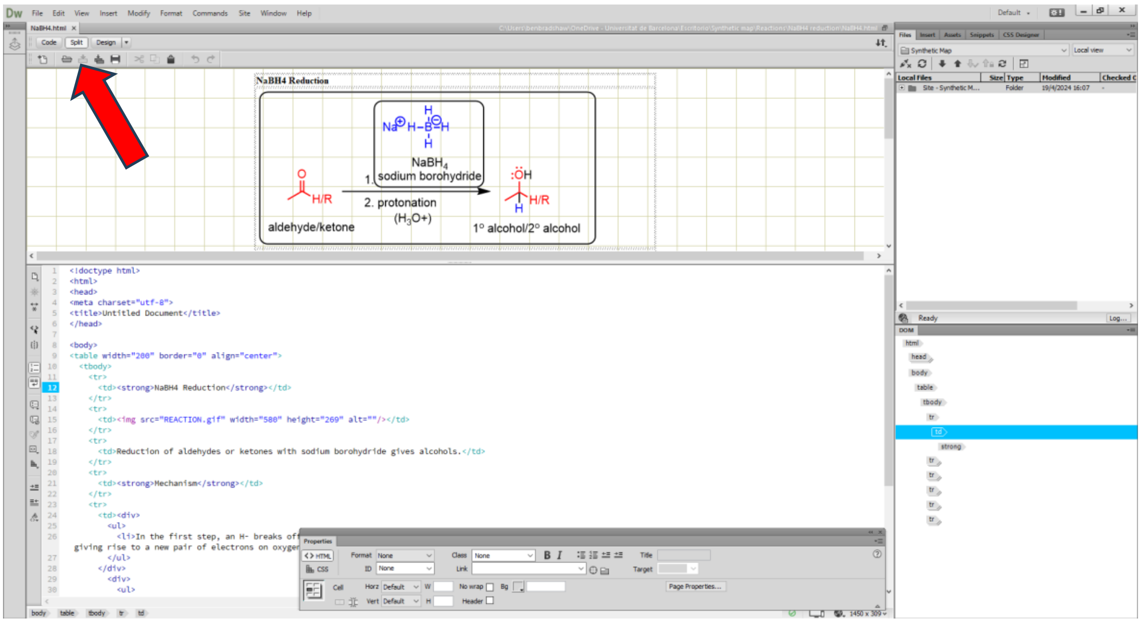

Add the HTML tags **^{** and **}** around any number or charge you want to display in superindex.

Use the HTML tags _{and} around the item to display in subindex format.

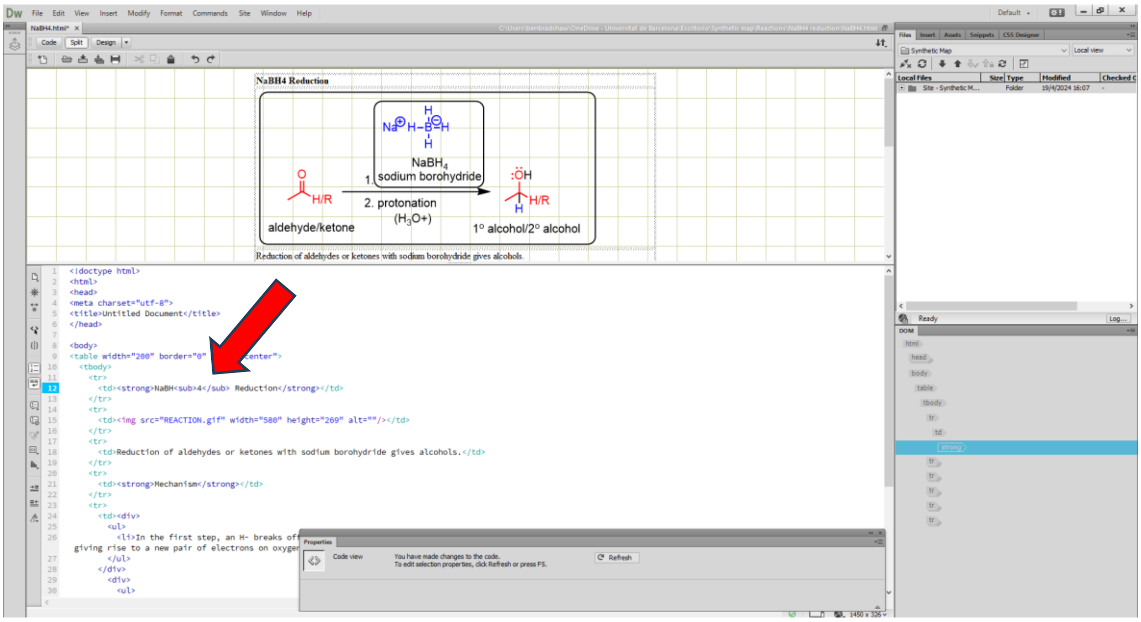

Finally, make any changes formatting changes by clicking on page properties to select the font and size.

For this example, we chose page font: Segoe,… and text size: 18

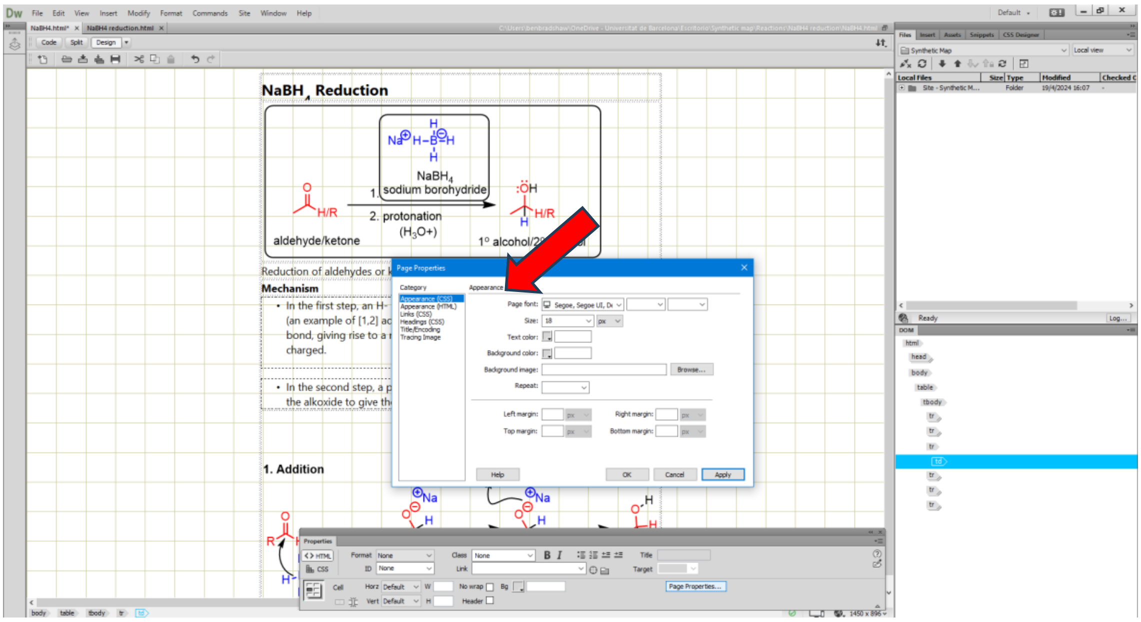

Repeat this process for each of the reactions in your map.

5. Link the reactions to the main page of the synthetic map

In the properties bar, select the circle option and outline one of the reactions.

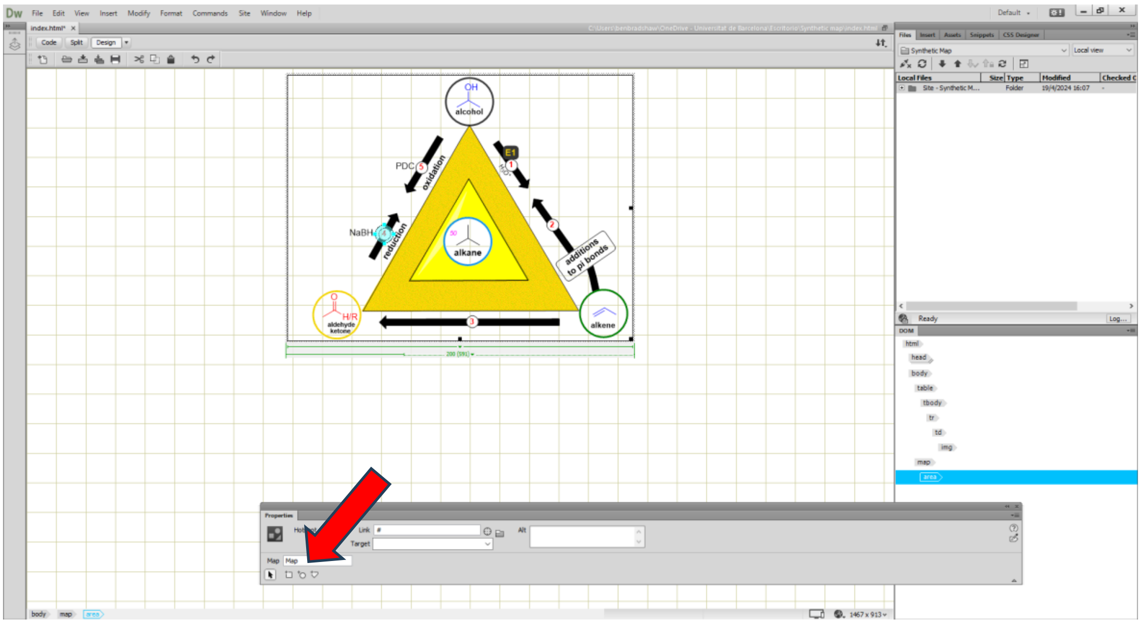

Then click on the blue circle and add where you want it to link. Here there are two possible options:

Option 1 - Link to a web address: Go to the web address of a page that contains information about the reaction. For example, you can link to the corresponding reaction page of our synthetic map by opening the page in your browser and copying the web address to the link box in the properties tab.

Option 2 - Link to a page you made in Dreamweaver in step 4: Click on the folder icon and find the corresponding HTML page in your synthetic map reactions folder.

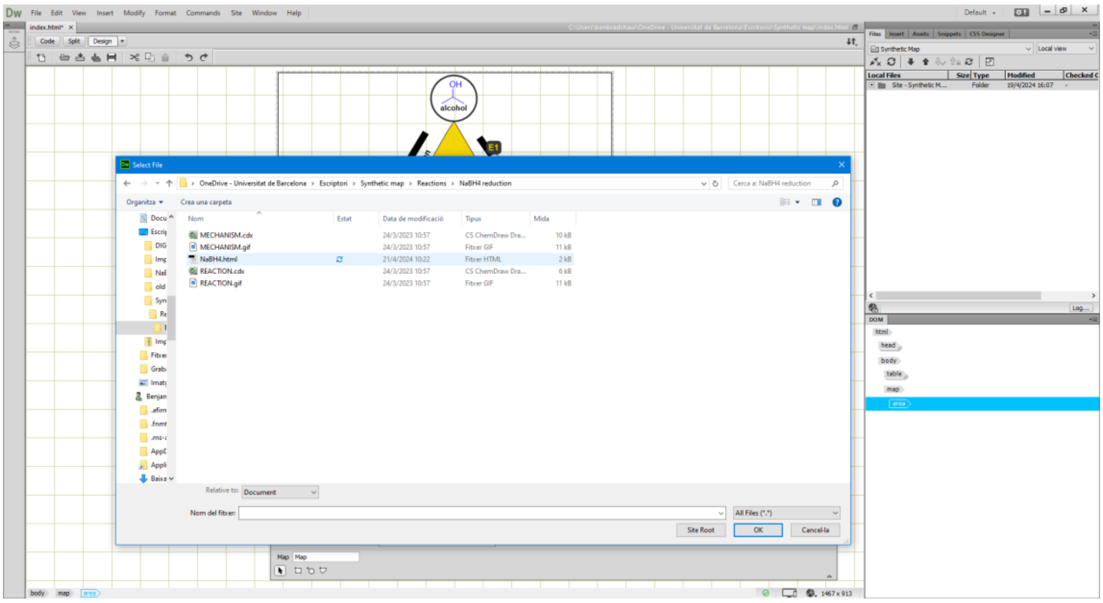

6. Upload the synthetic map to a server

Open the tab **Site** menu, add the name of your site, and indicate the location of the site folder.

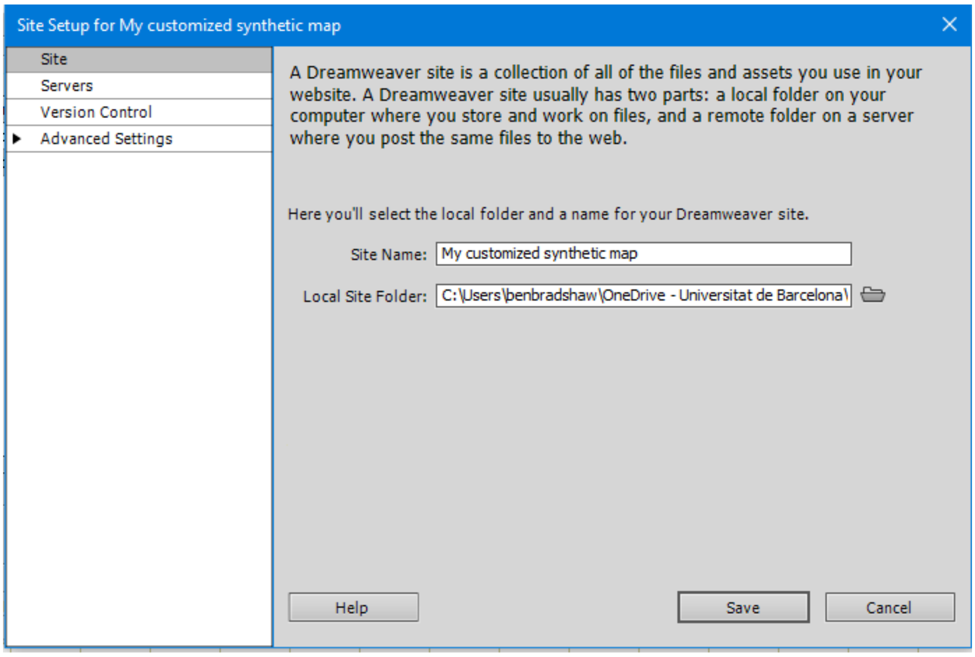

Then select the **Servers** tab and click on the **+** to add a new server.

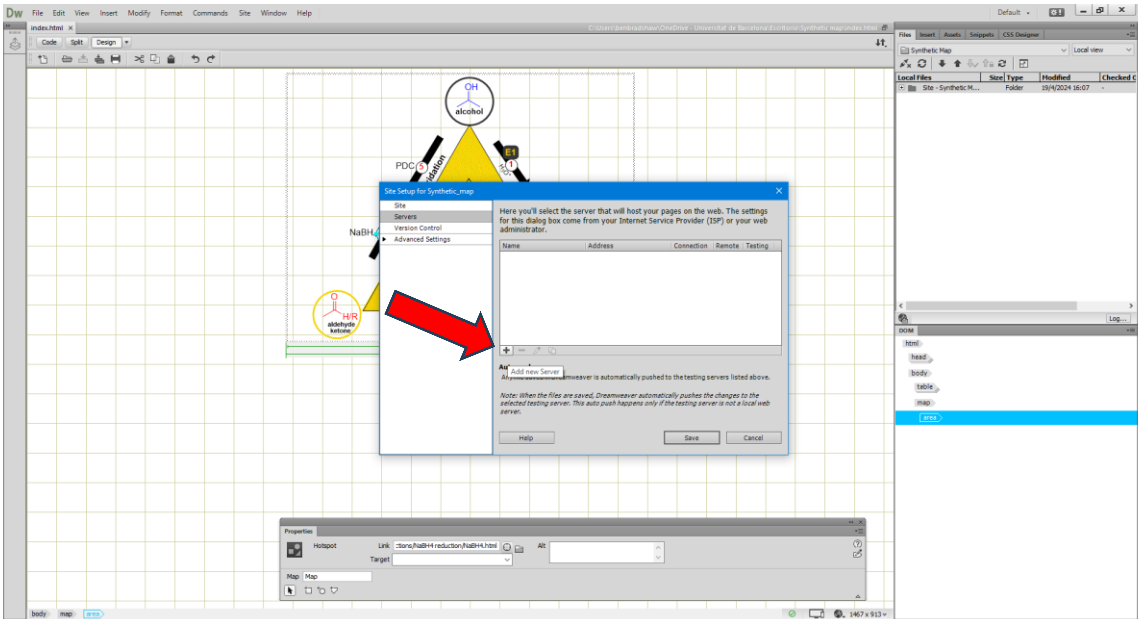

In the dialog box, fill in the required information. Your hosting provider will have provided you with the required codes. In the Web URL box, add your domain name linked to your hosting provider.

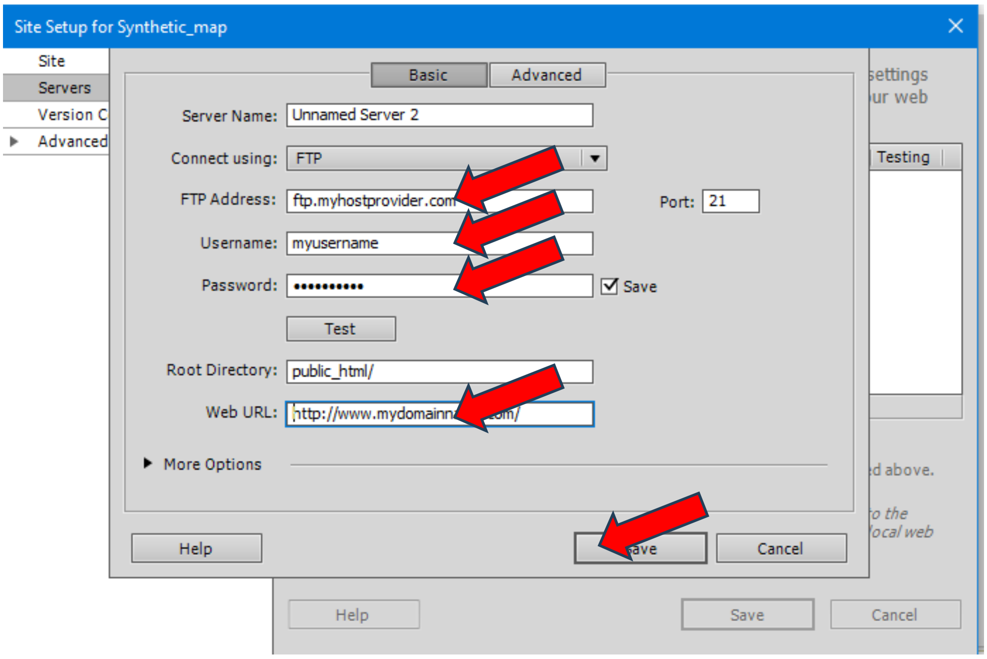

Now go to the right-hand tab of your files view – and press the ↑ arrow. Dreamweaver will upload your synthetic map to the hosting server.

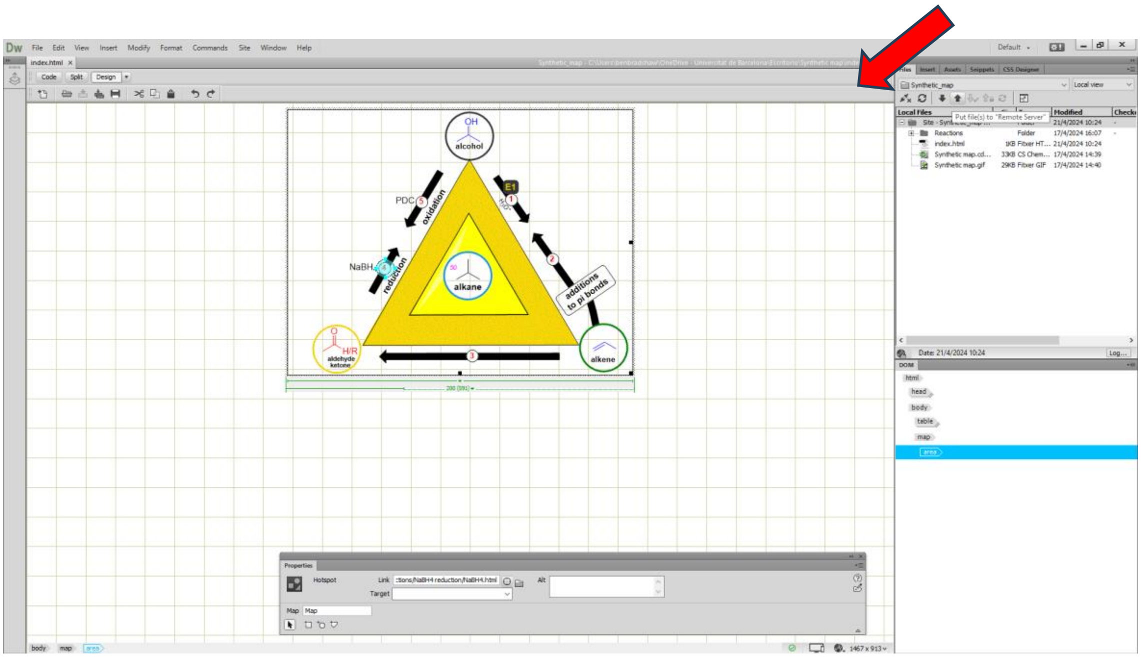

Supplement: Supplementary file 1 — ed4c00592_si_001.pdf [file ed4c00592_si_001.pdf]
